# Short-Term Findings From Testing EPIO, a Digital Self-Management Program for People Living With Chronic Pain: Randomized Controlled Trial

**DOI:** 10.2196/47284

**Published:** 2023-08-25

**Authors:** Katrine Bostrøm, Elin Børøsund, Hilde Eide, Cecilie Varsi, Ólöf Birna Kristjansdottir, Karlein M G Schreurs, Lori B Waxenberg, Karen E Weiss, Eleshia J Morrison, Hanne Stavenes Støle, Milada Cvancarova Småstuen, Audun Stubhaug, Lise Solberg Nes

**Affiliations:** 1 Department of Digital Health Research Division of Medicine Oslo University Hospital Oslo Norway; 2 Institute of Clinical Medicine Faculty of Medicine University of Oslo Oslo Norway; 3 Department of Nursing and Health Sciences Faculty of Health and Social Sciences University of South-Eastern Norway Drammen Norway; 4 Centre for Health and Technology Faculty of Health and Social Sciences University of South-Eastern Norway Drammen Norway; 5 Faculty of Health and Social Sciences University of South-Eastern Norway Drammen Norway; 6 Norwegian National Advisory Unit on Learning and Mastery in Health Oslo University Hospital Oslo Norway; 7 Mental Health Team West Primary Care of the Capital area Reykjavik Iceland; 8 Department of Psychology Health & Technology University of Twente Enschede Netherlands; 9 Department of Clinical and Health Psychology College of Public Health and Health Professions University of Florida Gainesville, FL United States; 10 Department of Psychiatry and Psychology College of Medicine and Science Mayo Clinic Rochester, MN United States; 11 Department of Child Health and Development Norwegian Institute of Public Health Oslo Norway; 12 Faculty of Health Sciences Oslo Metropolitan University Oslo Norway; 13 Department of Pain Management and Research Oslo University Hospital Oslo Norway; 14 Regional Advisory Unit on Pain Oslo University Hospital Oslo Norway

**Keywords:** chronic pain, self-management, digital health, efficacy, cognitive behavioral therapy, acceptance and commitment therapy

## Abstract

**Background:**

Chronic pain conditions involve numerous physical and psychological challenges, and while psychosocial self-management interventions can be of benefit for people living with chronic pain, such in-person treatment is not always accessible. Digital self-management approaches could improve this disparity, potentially bolstering outreach and providing easy, relatively low-cost access to pain self-management interventions.

**Objective:**

This randomized controlled trial aimed to evaluate the short-term efficacy of EPIO (ie, inspired by the Greek goddess for the soothing of pain, Epione), a digital self-management intervention, for people living with chronic pain.

**Methods:**

Patients (N=266) were randomly assigned to either the EPIO intervention (n=132) or a care-as-usual control group (n=134). Outcome measures included *pain interference* (Brief Pain Inventory; primary outcome measure), *anxiety* and *depression* (Hospital Anxiety and Depression Scale), *self-regulatory fatigue* (Self-Regulatory Fatigue 18 scale), *health-related quality of life* (SF-36 Short Form Health Survey), *pain catastrophizing* (Pain Catastrophizing Scale), and *pain acceptance* (Chronic Pain Acceptance Questionnaire). Linear regression models used change scores as the dependent variables.

**Results:**

The participants were primarily female (210/259, 81.1%), with a median age of 49 (range 22-78) years and a variety of pain conditions. Analyses (n=229) after 3 months revealed no statistically significant changes for the primary outcome of pain interference (*P*=.84), but significant reductions in the secondary outcomes of depression (mean difference −0.90; *P*=.03) and self-regulatory fatigue (mean difference −2.76; *P*=.008) in favor of the intervention group. No other statistically significant changes were observed at 3 months (all *P*>.05). Participants described EPIO as useful (ie, totally agree or agree; 95/109, 87.2%) and easy to use (101/109, 92.7%), with easily understandable exercises (106/109, 97.2%).

**Conclusions:**

Evidence-informed, user-centered digital pain self-management interventions such as EPIO may have the potential to effectively support self-management and improve psychological functioning in the form of reduced symptoms of depression and improved capacity to regulate thoughts, feelings, and behavior for people living with chronic pain.

**Trial Registration:**

ClinicalTrials.gov NCT03705104; https://clinicaltrials.gov/ct2/show/NCT03705104

## Introduction

### Background

Chronic pain conditions are common and can be disabling and distressing [1,2]. People living with chronic pain face daily challenges related to symptom management, activities, roles, and relationships, and coping with the emotional and sometimes cognitive impact of pain can be taxing. The many demands of living with chronic pain may also affect the ability to self-regulate (ie, regulate thoughts, emotions, and behavior) [3-5]. Given the complexity of chronic pain and living with chronic pain, a multidisciplinary treatment approach for pain management, aimed at supporting people in coping with physiological as well as psychological challenges, is recommended [2,6,7].

Psychological treatment approaches such as cognitive behavioral therapy (CBT) [8,9] and acceptance and commitment therapy (ACT) [10,11] have shown to be beneficial for pain across classification and pain etiology, with links to reductions in symptoms of pain, distress, depression, and anxiety, as well as improved quality of life and pain acceptance [12,13]. Regrettably, such evidence-based in-person psychological treatments are not always offered or accessible [7,14], with barriers to treatment including availability, financial costs, distance to treatment site, transportation issues, and personal preferences [15,16]. The limited availability of in-person psychological interventions for chronic pain, combined with individual barriers to engagement with such interventions, highlights the need to expand the care and delivery of psychosocial support for people living with chronic pain.

Digital solutions may have the potential to improve outreach and provide easy and relatively low-cost access to pain self-management interventions [17,18]. Existing digital pain self-management interventions have, for example, shown promising results in meeting unfulfilled needs, supporting psychological well-being, and improving health-related quality of life (HRQoL) [17-20]. However, there are several challenges with the existing digital pain management programs. Most such programs appear to focus on providing information about pain and collecting and monitoring data (eg, tracking medication use) and often lack a theoretical basis, such as evidence-based psychosocial interventional aspects [21,22]. Many existing digital interventions for pain self-management also lack user-centeredness, with the limited involvement of end users (ie, people with chronic pain) and health care providers in the development process [21,23,24]. In addition, attrition has surfaced as a serious challenge with digital interventions, highlighting the need for ways to bolster intervention adherence when designing and developing these interventions [25,26]. Finally, rigorous efficacy testing to establish efficacy appears to be scarce in the existing digital pain self-management interventions [17,27], with subsequent limited evidence of actual implementation into clinical care after study completion [22,28].

In response to the identified limitations in existing digital pain self-management interventions, we designed, developed, and feasibility pilot-tested EPIO (ie, inspired by the Greek goddess for the soothing of pain, Epione), a digital pain self-management intervention aimed at supporting patients living with chronic pain [29-33]. Although the type, form, and intensity of pain may differ depending on the condition and diagnosis (eg, neck or back pain, fibromyalgia, or trigeminal neuralgia) and other individual factors, EPIO was developed aiming to target chronic pain *in general* (ie, across pain classification and etiology) based on user input and the CBT- and ACT-based treatment approaches for people living with chronic pain. The EPIO intervention was designed and developed in close collaboration with researchers, eHealth experts, end users, and health care providers [29-31].

In line with recommendations from the Medical Research Council (MRC) framework for the evaluation of complex interventions [34,35], a feasibility pilot study was conducted, identifying EPIO as useful and easy to use, with excellent user satisfaction [32]. In line with the MRC guidelines, a qualitative study examining participants’ experiences when engaging with EPIO complemented the feasibility pilot findings, pointing to engagement factors such as the motivation to learn, fostering joy and enthusiasm, and personalization, as well as factors related to coping with pain in everyday life (eg, awareness and need for practice) and the value of engaging with EPIO (eg, EPIO—a friend and making peace with the presence of pain) [33]. Program optimization based on user feedback [32,33] was conducted before embarking on efficacy testing in a randomized controlled trial (RCT).

### Objectives

This study aimed to examine short-term (ie, 3 months) efficacy findings from an RCT testing the digital pain self-management intervention program EPIO. It was hypothesized that participants receiving EPIO, compared with participants in a care-as-usual control group, would experience significant improvements in primary (ie, pain interference) and secondary (ie, depression, anxiety, self-regulatory fatigue, HRQoL, pain catastrophizing, and pain acceptance) outcomes after 3 months of access to the EPIO program. System use, usefulness, and ease of use were examined on an exploratory basis.

## Methods

### Study Design

A 2-armed RCT was used with participants randomly assigned to (1) the digital pain self-management intervention program EPIO or (2) a care-as-usual control group.

### Participants and Recruitment

Participants were people living with chronic pain, recruited from November 2019 to February 2021 through a major medical institution (Oslo University Hospital, Norway), collaborating with local health care services and primary care practices, social media channels, and patient organizations’ web pages. The eligibility criteria were as follows: (1) living with chronic pain in general (ie, across pain classification and etiology); (2) pain duration ≥3 months (ie, self-reported); (3) age ≥18 years; (4) access to a smartphone or tablet; (5) ability to understand oral and written Norwegian; and (6) ability to attend an in-person introduction session either at a nearby health care facility or by using a secure video link (ie, due to pandemic restrictions as of spring 2020). The exclusion criteria included cancer-related pain, migraine, and untreated severe psychological illness (eg, psychosis), all of which were self-reported.

### Study Procedure

Patients living with chronic pain were verbally informed about the EPIO study by collaborating partners or through flyers at various health care sites. If interested, the patient’s contact information was forwarded to the project team, who then provided additional information about study participation to those interested. Patients could also contact the study staff directly through a study phone number or website. Study information was also published on social media (eg, Facebook), and these posts were frequently republished by individuals or patient organizations.

All participants provided written informed consent before completing the baseline outcome measures through a secure research server at the Services for Sensitive Data (*Tjenester for Sensitive Data* [TSD]; University of Oslo). Randomization, either to the EPIO intervention or the care-as-usual control group, was computerized (ie, using the R-tool software program, locally developed by the Department of Digital Health Research at Oslo University Hospital) and stratified by sex, with study arms 1:1 and a block size of 20. As participants were either assigned to receive the EPIO intervention or not, participation could not be blinded once group assignment was completed.

The intervention group participated in a face-to-face introduction session where they received (1) an introduction to the EPIO intervention program, (2) help to download the EPIO app from the Apple App Store or Google Play Store, and (3) guidance on how to use the program. The introduction session was initially planned to be an in-person session but was also provided digitally (ie, videoconference) as of spring 2020 due to the ongoing COVID-19 pandemic.

The study period was 3 months after the introduction session. The intervention group received follow-up phone calls from the members of the project team at approximately 3 and 7 weeks with standard questions about status (eg, to see how the use of the EPIO program was going and whether they had any use-related questions). The project team could also be contacted through the project study phone for questions or technical assistance. System use was logged, and outcome measures were completed through the secure research server. Participants completing at least 6 (67%) of the total 9 EPIO modules during the study period were defined as program completers for study purposes [32,36].

### The EPIO Intervention Program

**Figure 1:**
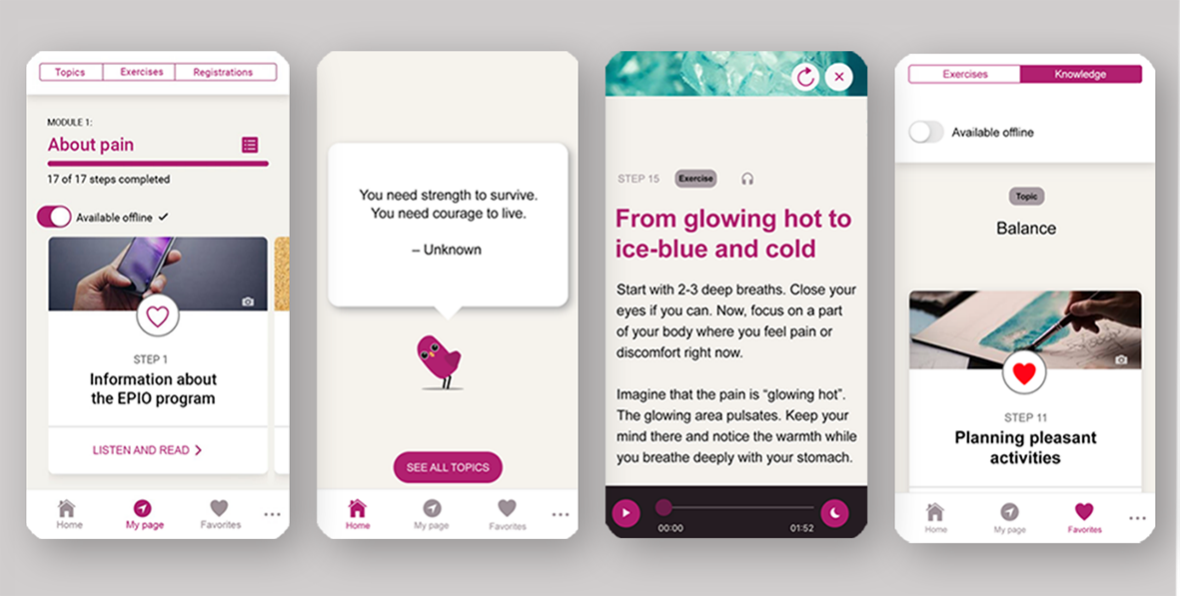
Example screenshots of the EPIO intervention program.

The app-based EPIO program consists of 9 CBT-based modules with aspects of ACT, each combining educational information (eg, thought challenges, coping strategies, values, and activity pacing) with practical and related exercises (eg, diaphragmatic breathing, progressive muscle relaxation, visualization, and mindfulness) for people living with chronic pain [30]. The 9 modules include information about (1) pain; (2) balance; (3) thoughts and feelings; (4) stress and coping; (5) what is important to me (ie, values); (6) behaviors and lifestyle; (7) communication, relations, and social support; (8) coping during difficult times; and (9) summary and the road ahead [30]. Through the EPIO program, progression is supported and guided by the EPIOS bird, an avatar *accompanying* the participants and their program progression. Participants are encouraged to practice content frequently to familiarize themselves with the psychoeducational content and exercises, and a 3-day *practice mode* delay has therefore been incorporated between modules (ie, from completing 1 module until the next module becomes available). The first 5 EPIO modules are sequential owing to the educational structure, while the participants can choose the order of modules 6 to 8 to allow for individualization. As participants proceed through the program, they can also create a list of favorites by highlighting exercises and topics, and they can opt to receive reminders based on their needs and preferences. EPIO also allows the user to choose between reading and listening or both, and there is an option to use the program offline. More detailed information about the design and development of the EPIO intervention program has been presented elsewhere [29-31]. See Figure 1 for screenshots of the EPIO program.

### Care-As-Usual Control Group

Participants in the care-as-usual control group completed the study outcome measures at baseline and at 3 months, without any additional follow-up from the research team. The project team did not seek to monitor or control any type of additional care potentially sought by participants in the care-as-usual control group during the study period. If interested, participants in the control group received access to the EPIO program after the completion of the study (ie, 12 months).

### Data Collection and Outcome Measures

All questionnaires and outcome measures were completed by the participants (ie, self-report) using the secure TSD server. Participants completed a study-specific sociodemographic and disease-related questionnaire at baseline, and outcome measures were collected at baseline (ie, before randomization) and at 3 months (ie, follow-up) after the introduction session.

#### Psychosocial Outcome Measures

##### Primary Outcome

*Pain interference* was measured using the short version of the Brief Pain Inventory (BPI) [37]. The BPI consists of 7 items and measures the impact of pain on daily functioning. The questionnaire has been validated in a Norwegian chronic pain population sample [38] and has acceptable internal consistency and reliability [37]. The BPI score ranges from 0 to 10, with higher scores indicating greater pain interference.

##### Secondary Outcomes

*Symptoms of anxiety and depression* were measured using the Hospital Anxiety and Depression Scale (HADS) [39]. The HADS consists of 14 items measuring anxiety (7 items) and depressive (7 items) symptomatology. The questionnaire has acceptable internal consistency and reliability and has been validated in Norwegian primary care patients and the general population [40]. The HADS scores range from 0 to 21 for both scales, with higher scores indicating a higher presence of symptoms of anxiety or depression.

*Self-regulatory fatigue* was measured using the Self-Regulatory Fatigue 18 scale [41]. The Self-Regulatory Fatigue 18 consists of 18 items measuring self-regulatory capacity with cognitive, emotional, and behavioral components. 8 items are positively phrased (eg, *I rarely get frustrated*, *I have no trouble making decisions*, and *I handle stress well*), and 10 items are negatively phrased (eg, *I experience repeated unpleasant thoughts*, *I feel moody*, and *I get easily upset*). The measure has been translated and tested in Norwegian cancer populations [42,43] and has acceptable internal consistency and reliability [41], and the score ranges from 18 to 90, with higher scores indicating higher self-regulatory fatigue.

*HRQoL* was assessed using the noncommercial SF-36 Short Form Health Survey (RAND-36) [44,45]. The RAND-36 consists of 36 items measuring physical, role, emotional, cognitive, and social functions as well as physical, general, and global health. The questionnaire has acceptable internal consistency and reliability and has been validated in a Norwegian population sample with chronic pain [45]. The RAND-36 scores range from 0 to 100, with higher subscale scores indicating improved HRQoL.

*Pain catastrophizing* was measured using the Pain Catastrophizing Scale (PCS) [46]. The PCS consists of 13 items measuring catastrophic thinking and maladaptive responses to pain, with 3 subscales measuring helplessness, magnification, and rumination. The questionnaire has acceptable internal consistency and reliability and has been validated in a Norwegian population sample with chronic pain [47]. The PCS score ranges from 0 to 52, with higher scores indicating a greater presence of catastrophic thoughts and feelings about pain.

*Pain acceptance* was measured using the short form of the Chronic Pain Acceptance Questionnaire (CPAQ)–8 [48]. The CPAQ-8 consists of 8 items measuring pain acceptance, with 4 items gauging pain willingness and the other 4 activity engagement [48]. The questionnaire has acceptable internal consistency and reliability and has been validated in a Norwegian population sample with chronic pain [49]. The CPAQ-8 score ranges from 0 to 24, with higher scores indicating a higher acceptance of pain.

#### System Use, Usefulness, and Ease of Use

Details related to use and program progress (ie, system use) were collected automatically, stored on a secure research server (ie, TSD), and later extracted in accordance with informed consent and existing safety and privacy regulations. At the 3-month follow-up, participants completed a 6-item study-specific questionnaire related to usefulness and ease of use based on the research team’s previous experience [30,42], inspired by Davis [50]. Participants completed 3 questions rating acceptability and feasibility on a scale from 1 to 5 (ranging from totally agree to totally disagree) and 3 open-ended questions regarding preferences, perceived usefulness, ease of use, and further suggestions. The System Usability Scale (SUS), examining system and program usability [50,51] on a 10-item 5-point scale with options ranging from strongly disagree to agree, was also completed at 3 months. The scores were summarized and multiplied by 2.5, yielding a value range of 0 to 100. Scores above 80.3 are considered excellent.

### Power Analysis and Sample Estimates

eHealth interventions for comparable samples have shown a Cohen *d* effect size of 0.30 to 0.40 on the primary outcome (ie, pain interference) [20,52]. On the basis of these findings, a sample size for the intervention effect on pain interference was calculated to allow the detection of Cohen *d*=0.4, with a Cronbach α value of .05 and 80% power (based on a 2 tailed *t* test). This study included 200 participants. To account for attrition and allow for adequate power in secondary outcome analyses, 266 participants were included.

### Statistical Analyses

Baseline characteristics, usefulness, ease of use, and user patterns were summarized with means and SDs for normally distributed variables and medians and ranges for variables with skewed distributions. The type of distribution was assessed using the visual inspection of histograms and q-q plots and by comparing means and medians. Categorical data were presented as counts and percentages. Change scores, defined as a difference between scores at baseline and at 3 months, were calculated for pain interference, anxiety, depression, self-regulatory fatigue, HRQoL, pain catastrophizing, and pain acceptance and used as dependent variables in linear regression models. As statistically significant differences were observed between the intervention and care-as-usual control groups for age and years living with pain, these variables were included in the intention-to-treat analysis as possible confounders. We included only individuals with data on both assessment points; thus, there were no missing data and no imputation of missing values was necessary. Model fit was tested using visual inspection of the residual plots of histograms, and the model fit was satisfactory for all the presented variables (ie, change scores). *P* values <.05 were considered statistically significant. The results are presented as estimated mean difference (MD) in change with 95% CI and effect size (ie, standardized coefficients β) [53]. All the presented CIs for model estimates were derived using bootstrapping with bias-corrected and accelerated correction (95% CI). Statistical analyses were performed using SPSS software (release 25; IBM Corp).

### Ethics Approval, Informed Consent, and Participation

The study was registered in ClinicalTrials.gov (NCT03705104) and approved by the Regional Committee for Medical and Health Research Ethics (REK 2018/8911) and the Hospital Privacy Protection Committee (ie, institutional review board equivalent; PVO 2017/6697). The study method and results were reported following the CONSORT-EHEALTH (Consolidated Standards of Reporting Trials of Electronic and Mobile Health Applications and Online Telehealth) checklist of information to include when reporting an RCT (Multimedia Appendix 1).

All potential participants received information about the nature of the study and details about study participation before deciding whether to participate. All study participants provided written informed consent. The signed consent forms were stored separately from any study data under lock and key in a separate departmental cabinet. Data from questionnaires and outcome measures were collected through the secure TSD platform throughout the study, and all personally identifiable information was deidentified from the TSD database before exporting the study data to a local secure server for further analysis. The participants did not receive compensation for participation in the study.

## Results

### Sample Description

**Figure 2:**
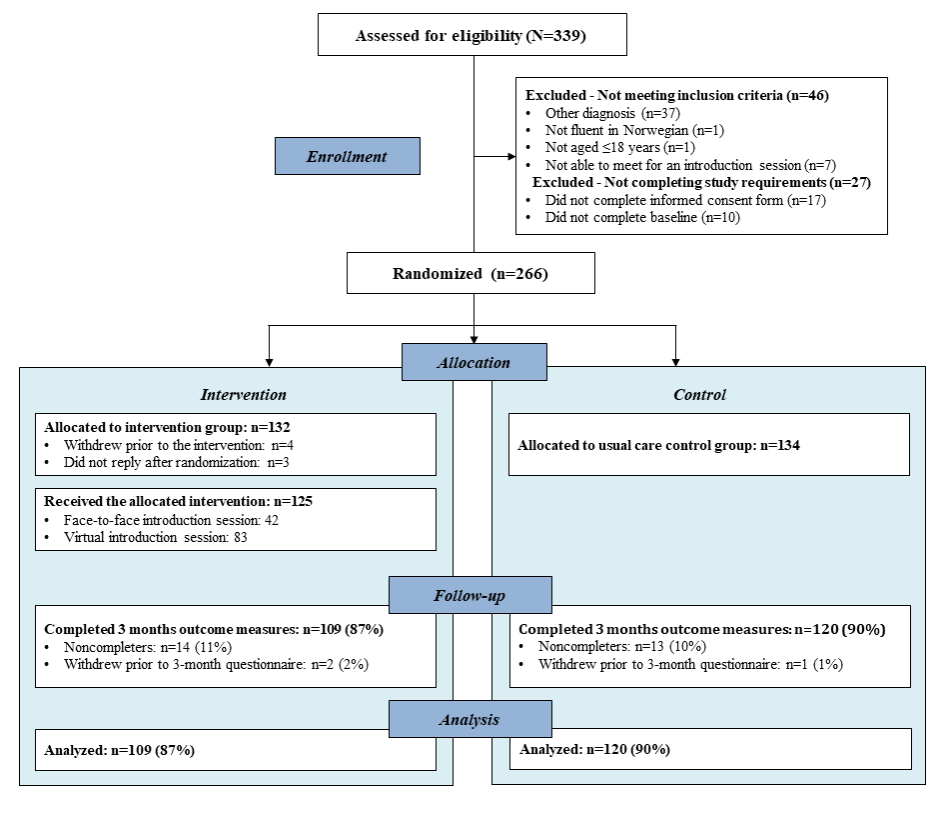
Recruitment and participant flow.

An overview of recruitment and retention details from baseline to the 3-month follow-up is shown in the trial recruitment and participant flowchart in Figure 2. A total of 266 participants living with chronic pain were enrolled in the study. In all, 7 participants allocated to the intervention group withdrew from the study or did not respond before the introduction session and were therefore excluded from the study. The final study sample was 259, with 125 participants in the intervention group and 134 in the control group.

Most participants (174/259, 67.2%) were recruited through social media, personally contacting the project via the study phone or website, and the remainder were recruited through collaboration with health care sites. The 259 participants were primarily White (n=251, 96.9%), with a median age of 49 (range 22-78) years at inclusion, and were mainly female (210/259, 81.1%). Most of the participants reported being on sick leave or disability benefits (186/259, 71.8%) at the time of enrollment and reported having lived with pain for ≥10 years (158/259, 61%). See Table 1 for an overview of baseline sociodemographic and disease-related characteristics.

There were statistically significant differences between the groups in terms of age (ie, intervention group participants were significantly older than those in the control group; 49.2 years vs 46.0 years, respectively; *P*=.02) and years lived with pain (ie, more participants in the intervention group reported having lived with pain for ≥10 years than those in the control group; 91 participants vs 72 participants, respectively; *P*=.007). There were no other statistically significant differences between the groups in terms of sociodemographic and disease-related variables (all *P*>.05). Of the 259 participants, 229 (88.4%) completed the outcome measures at the 3-month follow-up (intervention group, n=109, 47.6%; control group, n=120, 52.4%). See Figure 2 for details regarding the final number of participants. Of the 109 participants in the intervention group, 39 (35.8%) had received the introduction session in person and 70 (64.2%) received it through a secure video link owing to the COVID-19 pandemic restrictions.

**Table 1:** Baseline sociodemographic and disease-related characteristics (n=259).

Characteristics			Intervention (n=125)		Control (n=134)		*P* value	
Age (years), median (range)			50 (26-74)		48 (22-78)		.02	
**Sex, n (%)**								.64
	Female	103 (82.4)		107 (79.8)				
	Male	22 (17.6)		27 (20.1)				
**Marital status, n (%)**								.30
	Married or cohabitating	79 (63.2)		93 (69.4)				
	Single or divorced	46 (36.8)		41 (30.6)				
**Education, n (%)**								.49
	Elementary or high school	51 (40.8)		58 (43.3)				
	University or college <4 years	57 (45.6)		48 (35.8)				
	University or college >4 years	24 (19.2)		26 (19.4)				
**Employment, n (%)**								.58
	Full-time work or part-time work	30 (24)		27 (20.1)				
	Sick leave or disability benefits	86 (68.8)		100 (74.6)				
	Retired or others	9 (7.2)		7 (5.2)				
**Income status (€^a^), n (%)**								.92
	<40,000	28 (22.4)		26 (19.4)				
	>40,000 to 60,000	19 (15.2)		22 (16.4)				
	>60,000 to 80,000	32 (25.6)		31 (23.1)				
	>80,000 to 100,000	24 (19.2)		31 (23.1)				
	>100,000	22 (17.6)		24 (17.9)				
**Self-reported pain conditions^b^, n (%)**								
	Unspecific musculoskeletal pain	27 (21.6)		32 (23.9)		.77		
	Unspecified disk disorder	15 (12)		16 (11.9)		.93		
	Osteoarthritis	22 (17.6)		26 (19.4)		.87		
	Rheumatoid arthritis	16 (12.8)		13 (9.7)		.43		
	Fibromyalgia	52 (41.6)		54 (40.3)		.70		
	Neuropathic pain	10 (8)		9 (6.7)		.81		
	After injury or surgery	12 (9.6)		11 (8.2)		.58		
	Other	23 (18.4)		18 (13.4)		.16		
**Years living with pain, n (%)**								.03
	>3	12 (9.6)		22 (16.4)		.14		
	3-5	7 (5.6)		17 (12.7)		.06		
	5-10	20 (16)		23 (17.2)		.62		
	>10	91 (72.8)		72 (53.7)		.007		

^a^€1 is approximately US $1.1 and approximately 10 Norwegian kroner (as of spring 2022).

^b^Participants could report having several types of self-reported pain conditions.

### Group Differences

Primary and secondary outcomes, assessing between-group differences in changes from baseline to the 3-month follow-up, are reported in Multimedia Appendix 2. There was no statistically significant difference between the intervention and control groups in the primary outcome of pain interference on function at the 3-month follow-up (*P*=.84). However, there were significant reductions in symptoms of depression (ie, MD −0.90; *P*=.03) and self-regulatory fatigue (ie, MD −2.76; *P*=.008) in the EPIO intervention group compared with the care-as-usual control group. There were no statistically significant between-group differences in anxiety, pain acceptance, pain catastrophizing, or HRQoL at 3 months (all *P*>.05); a trend was observed for the HRQoL subscale of vitality (MD 4.25; *P*=.05) and the Chronic Pain Acceptance Item of Activity Engagement (MD 0.08; *P*=.08; Multimedia Appendix 2), but these results were not statistically significant.

### System Use, Usefulness, and Ease of Use

In total, 63 of 125 (50.4%) participants in the intervention group completed at least 6 of the 9 modules within the 3-month study period and were considered completers. There were no significant differences in sociodemographic variables or outcome measures (ie, MD in change) between program completers and noncompleters (all *P*>.05). Participants (n=109) completing outcome measures at the 3-month follow-up described the program as useful (ie, totally agree or agree; 95/109, 87.2%) and easy to use (101/109, 92.7%) and as having easily understandable exercises (106/109, 97.2%). Participants particularly reported appreciating the exercises (eg, diaphragmatic breathing), the combination of theory and interconnected exercises, encouraging messages (eg, set reminders), and the functionality of being able to choose between listening and reading. The median system usability (ie, SUS) score was 92.5 (range 32.5-100), indicating excellent (ie, grade A; SUS score >80.3) system usability.

## Discussion

### Principal Findings

In this RCT, people with chronic pain having access to the digital pain self-management intervention EPIO, compared with care-as-usual controls, did not report statistically significantly different changes in the primary outcome of pain interference on function after 3 months nor in the secondary outcomes of anxiety, HRQoL, pain catastrophizing, or pain acceptance. However, participants in the intervention group reported a significant decrease in symptoms of *depression* and *self-regulatory fatigue* compared with care-as-usual controls, indicating that people living with chronic pain may benefit from having access to evidence-informed, user-centered digital self-management programs, such as EPIO, specifically in terms of improving mood and self-regulation in the context of chronic pain.

With *pain* not being a single entity, rather a subjective, individual experience with substantial heterogeneity (eg, depending on the pain condition and diagnosis, individual, available treatment, and culture), finding adequate pain treatment and conducting successful pain trials have long been a conundrum [54,55]. The fact that this short-term RCT did not show statistical significance for the primary outcome of pain interference on function does not necessarily mean that there was no impact on pain-related aspects in the intervention group. Although it is a validated and reliable multidimensional instrument, the global estimates for the BPI [37] may not be sensitive enough to assess the interference of pain (ie, general activity, walking, work, mood, enjoyment of life, relations with others, and sleep) in a heterogeneous study population, as in this study. Effect sizes in this study were also small (β<.2), even with statistical significance, but the data variability was large, which may indicate that although some participants did not seem to benefit from EPIO with respect to all outcome measures, others may have benefited greatly from the intervention. Interventions can also have clinical importance despite not reaching statistical significance, and aspects such as data variability and sample size may influence statistical results [56].

The numerous physical and psychosocial challenges associated with living with chronic pain may naturally contribute to symptoms of *depression* [57,58]. In-person psychosocial interventions, particularly CBT-based interventions, can be effective in helping decrease the symptoms of depression [59], and this is also the case for CBT- and ACT-based interventions for people living with chronic pain [8,13]. Recently, digital CBT-based self-management programs have also shown promise in positively impacting depression [60,61]. The CBT-based content with aspects of ACT in EPIO [30] may as such have contributed to much of the significant decrease in symptoms of depression in the intervention group in this study. It is also possible that the way the EPIO program has been designed in line with existing recommendations [23,24], based on end-user and stakeholder input [30], plays a role in the perception, use, and ultimate effect of the program. The blended care delivery model used in this study, although simple, promoting knowledge that the research team is only one phone call or text message away, could also have provided a sense of connectedness with the research team for the participants [62,63]. This was also underlined when examining aspects of engagement with EPIO, with participants describing the EPIO program as fostering communication and social support [33].

Participants receiving EPIO, compared with participants in the care-as-usual control group, also reported a statistically significant decrease in *self-regulatory fatigue* after 3 months of use. Self-regulation, that is, a person’s ability to control or regulate cognitions, emotions, behavior, and to some extent physiology, is a vital part of life and *the self* [64,65]. However, depending on demands (eg, situational and environmental), self-regulatory capacity appears to be a limited resource that may be depleted or fatigued [65,66]. People living with chronic pain conditions face several physical (eg, pain and fatigue), behavioral (eg, overdoing or underdoing activities), cognitive (eg, rumination and worry), and emotional (eg, anxiety and depression) challenges, all of which entail a need for self-control or self-regulation [3], and people living with chronic pain conditions may, because of their condition, be susceptible to self-regulatory fatigue, perhaps even persistent self-regulatory fatigue [4].

Considering the possibility that self-regulatory fatigue may be a *missing link* in comprehending the complex aspects of chronic pain conditions [5], finding ways to improve self-regulatory capacity is vital [3-5,67]. This could potentially be done by aiming to target areas such as stress management, cognitive behavioral aspects, physical activity, nutrition, and sleep [5,68]. The CBT- and ACT-based EPIO intervention contains a balance between educational (eg, coping, problem solving, thought challenges, and activity pacing) and relaxation-type (eg, diaphragmatic breathing, progressive muscle relaxation, and visualization) exercises [30]. Therefore, it is possible that the EPIO intervention through content does target self-regulation. Other CBT-based digital interventions combining educational and relaxation-based content, tested in other patient groups, support this notion, showing positive self-regulatory impact [42,43].

At the 3-month follow-up, half of the participants had completed at least 6 out of 9 modules, slightly below the completion rate in the EPIO feasibility pilot study (62%) [32]. It is possible that the in-person introduction sessions (ie, 100% in the feasibility pilot study vs 34% in this RCT) could have contributed to the differences in completion or adherence between the 2 studies. However, a comparison between participants receiving the introduction session in person (42/125, 33.6%) versus via video (83/125, 66.4%) in this study could not be justified as the change in delivery method was solely due to the onset of the COVID-19 pandemic. It is also possible that 3 months may not be enough time for participants living with chronic pain to complete the program, particularly as they were all instructed to spend as much time as possible practicing the module content in this study, rather than trying to complete the program quickly. In addition, even though the completion rate in this study may appear relatively low, 50% seems to be an average adherence rate for eHealth interventions [69], and program completion rates at 20% to 40% are not uncommon [70]. Further research is needed to examine whether access for longer periods (ie, >3 months) may help improve eHealth intervention adherence or whether other aspects, for example, additional follow-up or both, are called for.

There are several professional challenges in the transfer of traditional in-person service models to digital self-management interventions. In-person treatment entails several evident values, such as the therapist-patient relationship, but little is known about the type and frequency of human interaction that may be preferred or that may provide optimal effect. Reviews have indicated that professionally guided digital self-management interventions may be more effective than self-guided interventions [23,71] and that guided digital CBT-based programs may be more beneficial than unguided programs in terms of depression [72]. As such, the prioritization of human contact and exploration of increasingly personalized digital programs have been suggested [71]. It is possible that the simple blended care delivery in this study, combined with the avatar-like EPIOS bird guiding participants through the program, may have provided a certain combination of guidance and human contact. Blended care delivery, retaining advantages from in-person and digital solutions, may therefore be a recommended way to deliver such interventions in the future [23,33].

Finally, participants in this study described the EPIO program as useful (95/109, 87.2%) and easy to use (101/109, 92.7%), with easily understandable exercises (106/109, 97.2%) and excellent system usability (92.5 of 100). These findings are in line with those from the EPIO feasibility pilot study [32], with even higher system usability scores in this study (92.5 vs 85.7, respectively).

### Study Limitations, Strengths, and Future Directions

This study had several limitations. First, participants were recruited through social media and by collaborating with project partners, which may suggest that the study population consisted of highly motivated individuals. Therefore, it is not clear whether people with chronic pain would, in general, be interested in or benefit from a digital self-management intervention such as EPIO. However, therapeutic interventions are likely mainly effective if the participants are indeed interested in participating, which may support the notion that interested participants may be more likely to benefit from such self-management programs.

Second, most participants in this study were female, were White, had higher education, and had lived with chronic pain for many years. Randomization in this study was computerized and stratified by sex to ensure even sex group distribution, but future studies should seek to identify ways to include more balanced proportions of participants (eg, sex, ethnicity, education, and years with pain) in self-management interventions [73,74].

Third, the EPIO intervention was developed aiming to target chronic pain *in general* (ie, across pain classification and etiology) by providing educational information and related exercises based on CBT and aspects of ACT for pain self-management and targeting a broader range of aspects related to self-management skills. Given the heterogeneity of chronic pain (eg, neck or back pain, spinal cord injury, fibromyalgia, or trigeminal neuralgia), future research should seek to explore whether tailored self-management interventions could benefit from a more pain condition–specific approach, with a more rigorous definition of inclusion.

Fourth, the intervention group was compared with a care-as-usual control group, in which the participants did not receive the EPIO intervention program or any other care from the project team. However, as there was no way of controlling or assessing whether participants in the control group sought any other type of care (eg, self-management courses or mindfulness interventions) during the study period, there is a potential risk that this unknown factor could have impacted the study results. Given this challenge with rather pragmatic RCTs, inquiring about the potential additional external care received by members of the care-as-usual control group might have provided further details about this unknown factor.

Fifth, the study did not include a numerical primary outcome measure cut-off score for study inclusion, and it is possible that such a cut-off score (eg, only including participants providing a score above mild pain interference, such as >3 or >5) could have affected the primary outcome results of this study. However, with pain and the interference of pain being an individual and subjective experience, the rationale for excluding people living with chronic pain based on subjective scoring must be thoroughly considered before implementation.

Sixth, although the statistical power for the between-group effects was adequate, future studies may consider testing potential moderating effects, for example, related to baseline medical comorbidities, in larger study samples. Finally, this study explored short-term (ie, 3 months) findings from an RCT testing the EPIO intervention program. Future research should examine these long-term findings and follow-up. Qualitative explorations may also, in line with recommendations from the MRC [34,35], contribute to a better understanding of the impact and nuances of using pain self-management interventions [33,35].

The fact that the EPIO intervention program was designed and developed based on existing recommendations for digital interventions, including a solid theoretical foundation and development in close collaboration with end users and other stakeholders (eg, health care professionals), is a clear study strength. The simple blended care delivery model used in the study may also guide how such digital interventions could be delivered and implemented in the future, and future research should also aim to test and compare various delivery approaches (eg, blended care delivery vs digital self-management only). This type of research could add value to the understanding of what may constitute the optimal digital intervention and its delivery for those living with chronic pain and also identify the population groups, stages, or types of condition that may benefit from digital pain self-management.

### Conclusions

Digital pain self-management interventions, such as EPIO, delivered in a simple blended care model, may have the potential to support self-management and improve coping and psychological functioning for people living with chronic pain. Despite not showing a statistically significant impact on the primary outcome of pain interference on function after 3 months in this RCT, participants with chronic pain conditions having access to the EPIO intervention showed a statistically significant decrease in depressive symptoms and self-regulatory fatigue (ie, increased capacity to regulate thoughts, feelings, and behavior), compared with those in the care-as-usual control group. Therefore, long-term efficacy testing is warranted and in progress.

## Appendix

Multimedia Appendix 1 CONSORT-eHEALTH (Consolidated Standards of Reporting Trials of Electronic and Mobile Health Applications and Online Telehealth) checklist (V 1.6.1).

Multimedia Appendix 2 Estimates at both time points and between-group differences in the change from baseline to the 3-month follow-up.

## Abbreviations

ACT: acceptance and commitment therapy
BPI: Brief Pain Inventory
CBT: cognitive behavioral therapy
CONSORT-EHEALTH: Consolidated Standards of Reporting Trials of Electronic and Mobile Health Applications and Online Telehealth
CPAQ: Chronic Pain Acceptance Questionnaire
HADS: Hospital Anxiety and Depression Scale
HRQoL: health-related quality of life
MD: mean difference
MRC: Medical Research Council
PCS: Pain Catastrophizing Scale
RAND-36: SF-36 Short Form Health Survey
RCT: randomized controlled trial
SUS: System Usability Scale
TSD: Tjenester for Sensitive Data (Services for Sensitive Data)

## Data Availability

Data sets from this study are, due to the nature of patient-sensitive information, not available for public sharing through public archives or repositories. However, deidentified data from this study will be made available in accordance with institutional standards upon contacting the corresponding author.
