# Supplementary material for: Short-Term Findings From Testing EPIO, a Digital Self-Management Program for People Living With Chronic Pain: Randomized Controlled Trial
Source: J Med Internet Res. 2023 Aug 25;25:e47284. doi: 10.2196/47284 (PMC10492177; doi:10.2196/47284)
Supplement: Multimedia Appendix 1 [file jmir_v25i1e47284_app1.pdf]

CONSORT-EHEALTH (V 1.6.1) - Submission/Publication Form

The CONSORT-EHEALTH checklist is intended for authors of randomized trials evaluating web-based and Internet-based applications/interventions, including mobile interventions, electronic games (incl multiplayer games), social media, certain telehealth applications, and other interactive and/or networked electronic applications. Some of the items (e.g. all subitems under item 5 - description of the intervention) may also be applicable for other study designs.

The goal of the CONSORT EHEALTH checklist and guideline is to be
a) a guide for reporting for authors of RCTs,
b) to form a basis for appraisal of an ehealth trial (in terms of validity)

CONSORT-EHEALTH items/subitems are MANDATORY reporting items for studies published in the Journal of Medical Internet Research and other journals / scientific societies endorsing the checklist.

Items numbered 1., 2., 3., 4a., 4b etc are original CONSORT or CONSORT-NPT (non-pharmacologic treatment) items.
Items with Roman numerals (i., ii, iii, iv etc.) are CONSORT-EHEALTH extensions/clarifications.

As the CONSORT-EHEALTH checklist is still considered in a formative stage, we would ask that you also RATE ON A SCALE OF 1-5 how important/useful you feel each item is FOR THE PURPOSE OF THE CHECKLIST and reporting guideline (optional).

Mandatory reporting items are marked with a red *.
In the textboxes, either copy & paste the relevant sections from your manuscript into this form - please include any quotes from your manuscript in QUOTATION MARKS,
or answer directly by providing additional information not in the manuscript, or elaborating on why the item was not relevant for this study.

YOUR ANSWERS WILL BE PUBLISHED AS A SUPPLEMENTARY FILE TO YOUR PUBLICATION IN JMIR AND ARE CONSIDERED PART OF YOUR PUBLICATION (IF ACCEPTED).
Please fill in these questions diligently. Information will not be copyedited, so please use proper spelling and grammar, use correct capitalization, and avoid abbreviations.

DO NOT FORGET TO SAVE AS PDF _AND_ CLICK THE SUBMIT BUTTON SO YOUR ANSWERS ARE IN OUR DATABASE !!!

Citation Suggestion (if you append the pdf as Appendix we suggest to cite this paper in the caption):
Eysenbach G, CONSORT-EHEALTH Group [illegible]
[illegible] J Med Internet Res 2011;13(4):e126

Du endrer svaret ditt. Hvis du deler denne nettadressen, kan andre også endre svaret ditt.
FYLL UT ET NYTT SVAR

URL: http://www.jmir.org/2011/4/e126/
doi: 10.2196/jmir.1923
PMID: 22209829

katrine.bostrom@gmail.com (ikke delt) Bytt konto

Send inn på nytt for å lagre

* Må fylles ut

Your name *

First Last

Katrine Bostrøm

Primary Affiliation (short), City, Country *

University of Toronto, Toronto, Canada

Department of Digital Health, Division of Medic

Your e-mail address *

abc@gmail.com

katrine.bostrom@rr-research.no

Title of your manuscript *

Provide the (draft) title of your manuscript.

Short-term findings from a randomized controlled trial testing EPIO – a digital self-management program for people living with chronic pain

Du endrer svaret ditt. Hvis du deler denne nettadressen, kan andre også endre svaret ditt.

FYLL UT ET NYTT SVAR

Name of your App/Software/Intervention *

If there is a short and a long/alternate name, write the short name first and add the long name in brackets.

EPIO

Evaluated Version (if any)

e.g. "V1", "Release 2017-03-01", "Version 2.0.27913"

Svaret ditt

Language(s) *

What language is the intervention/app in? If multiple languages are available, separate by comma (e.g. "English, French")

Norwegian

URL of your Intervention Website or App

e.g. a direct link to the mobile app on app in appstore (itunes, Google Play), or URL of the website. If the intervention is a DVD or hardware, you can also link to an Amazon page.

Svaret ditt

URL of an image/screenshot (optional)

Svaret ditt

Du endrer svaret ditt. Hvis du deler denne nettadressen, kan andre også endre svaret ditt.

FYLL UT ET NYTT SVAR

Accessibility *

Can an enduser access the intervention presently?

- ○ access is free and open
- ○ access only for special usergroups, not open
- ◉ access is open to everyone, but requires payment/subscription/in-app purchases
- ○ app/intervention no longer accessible
- ○ Andre:

Primary Medical Indication/Disease/Condition *

e.g. "Stress", "Diabetes", or define the target group in brackets after the condition, e.g. "Autism (Parents of children with)", "Alzheimers (Informal Caregivers of)"

Chronic pain

Primary Outcomes measured in trial *

comma-separated list of primary outcomes reported in the trial

Pain interference

Secondary/other outcomes

Are there any other outcomes the intervention is expected to affect?

Depression, anxiety, self-regulatory fatigue, health-related quality of life (HRQoL), pain catastrophizing, and pain acceptance

Du endrer svaret ditt. Hvis du deler denne nettadressen, kan andre også endre svaret ditt.

FYLL UT ET NYTT SVAR

Recommended "Dose" *

What do the instructions for users say on how often the app should be used?

- ( ) Approximately Daily
- ( ) Approximately Weekly
- ( ) Approximately Monthly
- ( ) Approximately Yearly
- ( ) "as needed"
- ( ) Andre:

Approx. Percentage of Users (starters) still using the app as recommended after 3 months *

- ( ) unknown / not evaluated
- ( ) 0-10%
- ( ) 11-20%
- ( ) 21-30%
- ( ) 31-40%
- ( ) 41-50%
- ( ) 51-60%
- ( ) 61-70%
- ( ) 71%-80%
- ( ) 81-90%
- ( ) 91-100%
- ( ) Andre:

Du endrer svaret ditt. Hvis du deler denne nettadressen, kan andre også endre svaret ditt.

FYLL UT ET NYTT SVAR

Overall, was the app/intervention effective? *

- ○ yes: all primary outcomes were significantly better in intervention group vs control
- ○ partly: SOME primary outcomes were significantly better in intervention group vs control
- ○ no statistically significant difference between control and intervention
- ○ potentially harmful: control was significantly better than intervention in one or more outcomes
- ○ inconclusive: more research is needed
- ◉ Andre: Some secondary outcomes were significantly better in intervention gr

Article Preparation Status/Stage *

At which stage in your article preparation are you currently (at the time you fill in this form)

- ○ not submitted yet - in early draft status
- ◉ not submitted yet - in late draft status, just before submission
- ○ submitted to a journal but not reviewed yet
- ○ submitted to a journal and after receiving initial reviewer comments
- ○ submitted to a journal and accepted, but not published yet
- ○ published
- ○ Andre:

Du endrer svaret ditt. Hvis du deler denne nettadressen, kan andre også endre svaret ditt.

FYLL UT ET NYTT SVAR

Journal *

If you already know where you will submit this paper (or if it is already submitted), please provide the journal name (if it is not JMIR, provide the journal name under "other")

- ○ not submitted yet / unclear where I will submit this
- ◉ Journal of Medical Internet Research (JMIR)
- ○ JMIR mHealth and UHealth
- ○ JMIR Serious Games
- ○ JMIR Mental Health
- ○ JMIR Public Health
- ○ JMIR Formative Research
- ○ Other JMIR sister journal
- ○ Andre: ____________

Is this a full powered effectiveness trial or a pilot/feasibility trial? *

- ○ Pilot/feasibility
- ◉ Fully powered

Manuscript tracking number *

If this is a JMIR submission, please provide the manuscript tracking number under "other" (The ms tracking number can be found in the submission acknowledgement email, or when you login as author in JMIR. If the paper is already published in JMIR, then the ms tracking number is the four-digit number at the end of the DOI, to be found at the bottom of each published article in JMIR)

- ◉ no ms number (yet) / not (yet) submitted to / published in JMIR
- ○ Andre: ____________

Du endrer svaret ditt. Hvis du deler denne nettadressen, kan andre også endre svaret ditt.

FYLL UT ET NYTT SVAR

TITLE AND ABSTRACT

1a) TITLE: Identification as a randomized trial in the title

1a) Does your paper address CONSORT item 1a? *

I.e does the title contain the phrase "Randomized Controlled Trial"? (if not, explain the reason under "other")

- (x) yes
- ( ) Andre:

1a-i) Identify the mode of delivery in the title

Identify the mode of delivery. Preferably use “web-based” and/or “mobile” and/or “electronic game” in the title. Avoid ambiguous terms like “online”, “virtual”, “interactive”. Use “Internet-based” only if Intervention includes non-web-based Internet components (e.g. email), use “computer-based” or “electronic” only if offline products are used. Use “virtual” only in the context of “virtual reality” (3-D worlds). Use “online” only in the context of “online support groups”. Complement or substitute product names with broader terms for the class of products (such as “mobile” or “smart phone” instead of “iphone”), especially if the application runs on different platforms.

subitem not at all important

- 1 ( )
- 2 ( )
- 3 ( )
- 4 ( )
- 5 (x)

essential

Opphev valget

Du endrer svaret ditt. Hvis du deler denne nettadressen, kan andre også endre svaret ditt.

FYLL UT ET NYTT SVAR

Does your paper address subitem 1a-i? *

Copy and paste relevant sections from manuscript title (include quotes in quotation marks "like this" to indicate direct quotes from your manuscript), or elaborate on this item by providing additional information not in the ms, or briefly explain why the item is not applicable/relevant for your study

"a digital self-management program"

1a-ii) Non-web-based components or important co-interventions in title

Mention non-web-based components or important co-interventions in title, if any (e.g., “with telephone support”).

subitem not at all important

1 ○

2 ○

3 ○

4 ○

5 ○

essential

Does your paper address subitem 1a-ii?

Copy and paste relevant sections from manuscript title (include quotes in quotation marks "like this" to indicate direct quotes from your manuscript), or elaborate on this item by providing additional information not in the ms, or briefly explain why the item is not applicable/relevant for your study

Svaret ditt

Du endrer svaret ditt. Hvis du deler denne nettadressen, kan andre også endre svaret ditt.

FYLL UT ET NYTT SVAR

1a-iii) Primary condition or target group in the title

Mention primary condition or target group in the title, if any (e.g., “for children with Type I Diabetes”) Example: A Web-based and Mobile Intervention with Telephone Support for Children with Type I Diabetes: Randomized Controlled Trial

subitem not at all important

1 ○

2 ○

3 ○

4 ◉

5 ○

essential

Opphev valget

Does your paper address subitem 1a-iii? *

Copy and paste relevant sections from manuscript title (include quotes in quotation marks "like this" to indicate direct quotes from your manuscript), or elaborate on this item by providing additional information not in the ms, or briefly explain why the item is not applicable/relevant for your study

"for people living with chronic pain"

1b) ABSTRACT: Structured summary of trial design, methods, results, and conclusions

NPT extension: Description of experimental treatment, comparator, care providers, centers, and blinding status.

Du endrer svaret ditt. Hvis du deler denne nettadressen, kan andre også endre svaret ditt.

FYLL UT ET NYTT SVAR

1b-i) Key features/functionalities/components of the intervention and comparator in the METHODS section of the ABSTRACT

Mention key features/functionalities/components of the intervention and comparator in the abstract. If possible, also mention theories and principles used for designing the site. Keep in mind the needs of systematic reviewers and indexers by including important synonyms. (Note: Only report in the abstract what the main paper is reporting. If this information is missing from the main body of text, consider adding it)

subitem not at all important

1 ○

2 ○

3 ○

4 ◉

5 ○

essential

Opphev valget

Does your paper address subitem 1b-i? *

Copy and paste relevant sections from the manuscript abstract (include quotes in quotation marks "like this" to indicate direct quotes from your manuscript), or elaborate on this item by providing additional information not in the ms, or briefly explain why the item is not applicable/relevant for your study

Patients (N=266) were randomly assigned to the EPIO intervention (n=132) or a care-as-usual control group (n=134).

Du endrer svaret ditt. Hvis du deler denne nettadressen, kan andre også endre svaret ditt.

FYLL UT ET NYTT SVAR

1b-ii) Level of human involvement in the METHODS section of the ABSTRACT

Clarify the level of human involvement in the abstract, e.g., use phrases like “fully automated” vs. “therapist/nurse/care provider/physician-assisted” (mention number and expertise of providers involved, if any). (Note: Only report in the abstract what the main paper is reporting. If this information is missing from the main body of text, consider adding it)

subitem not at all important

1 ◯

2 ◯

3 ◯

4 ◯

5 ◯

essential

Does your paper address subitem 1b-ii?

Copy and paste relevant sections from the manuscript abstract (include quotes in quotation marks "like this" to indicate direct quotes from your manuscript), or elaborate on this item by providing additional information not in the ms, or briefly explain why the item is not applicable/relevant for your study

Svaret ditt

Du endrer svaret ditt. Hvis du deler denne nettadressen, kan andre også endre svaret ditt.

FYLL UT ET NYTT SVAR

1b-iii) Open vs. closed, web-based (self-assessment) vs. face-to-face assessments in the METHODS section of the ABSTRACT

Mention how participants were recruited (online vs. offline), e.g., from an open access website or from a clinic or a closed online user group (closed usergroup trial), and clarify if this was a purely web-based trial, or there were face-to-face components (as part of the intervention or for assessment). Clearly say if outcomes were self-assessed through questionnaires (as common in web-based trials). Note: In traditional offline trials, an open trial (open-label trial) is a type of clinical trial in which both the researchers and participants know which treatment is being administered. To avoid confusion, use “blinded” or “unblinded” to indicated the level of blinding instead of “open”, as “open” in web-based trials usually refers to “open access” (i.e. participants can self-enrol). (Note: Only report in the abstract what the main paper is reporting. If this information is missing from the main body of text, consider adding it)

subitem not at all important

1 ○

2 ○

3 ○

4 ○

5 ○

essential

Does your paper address subitem 1b-iii?

Copy and paste relevant sections from the manuscript abstract (include quotes in quotation marks "like this" to indicate direct quotes from your manuscript), or elaborate on this item by providing additional information not in the ms, or briefly explain why the item is not applicable/relevant for your study

Svaret ditt

Du endrer svaret ditt. Hvis du deler denne nettadressen, kan andre også endre svaret ditt.

FYLL UT ET NYTT SVAR

1b-iv) RESULTS section in abstract must contain use data

Report number of participants enrolled/assessed in each group, the use/uptake of the intervention (e.g., attrition/adherence metrics, use over time, number of logins etc.), in addition to primary/secondary outcomes. (Note: Only report in the abstract what the main paper is reporting. If this information is missing from the main body of text, consider adding it)

subitem not at all important

1 ○

2 ○

3 ○

4 ○

5 ○

essential

Does your paper address subitem 1b-iv?

Copy and paste relevant sections from the manuscript abstract (include quotes in quotation marks "like this" to indicate direct quotes from your manuscript), or elaborate on this item by providing additional information not in the ms, or briefly explain why the item is not applicable/relevant for your study

Svaret ditt

Du endrer svaret ditt. Hvis du deler denne nettadressen, kan andre også endre svaret ditt.

FYLL UT ET NYTT SVAR

1b-v) CONCLUSIONS/DISCUSSION in abstract for negative trials

Conclusions/Discussions in abstract for negative trials: Discuss the primary outcome - if the trial is negative (primary outcome not changed), and the intervention was not used, discuss whether negative results are attributable to lack of uptake and discuss reasons. (Note: Only report in the abstract what the main paper is reporting. If this information is missing from the main body of text, consider adding it)

subitem not at all important

1 ○

2 ○

3 ○

4 ○

5 ○

essential

Does your paper address subitem 1b-v?

Copy and paste relevant sections from the manuscript abstract (include quotes in quotation marks "like this" to indicate direct quotes from your manuscript), or elaborate on this item by providing additional information not in the ms, or briefly explain why the item is not applicable/relevant for your study

Svaret ditt

INTRODUCTION

2a) In INTRODUCTION: Scientific background and explanation of rationale

Du endrer svaret ditt. Hvis du deler denne nettadressen, kan andre også endre svaret ditt.

FYLL UT ET NYTT SVAR

2a-i) Problem and the type of system/solution

Describe the problem and the type of system/solution that is object of the study: intended as stand-alone intervention vs. incorporated in broader health care program? Intended for a particular patient population? Goals of the intervention, e.g., being more cost-effective to other interventions, replace or complement other solutions? (Note: Details about the intervention are provided in "Methods" under 5)

subitem not at all important

1 ○

2 ○

3 ○

4 ◉

5 ○

essential

Opphev valget

Does your paper address subitem 2a-i? *

Copy and paste relevant sections from the manuscript (include quotes in quotation marks "like this" to indicate direct quotes from your manuscript), or elaborate on this item by providing additional information not in the ms, or briefly explain why the item is not applicable/relevant for your study

In response to identified limitations with existing digital pain self-management interventions, the current research team designed, developed and feasibility pilot tested EPIO, a digital pain self-management intervention aiming to support patients living with chronic pain.

Du endrer svaret ditt. Hvis du deler denne nettadressen, kan andre også endre svaret ditt.

FYLL UT ET NYTT SVAR

2a-ii) Scientific background, rationale: What is known about the (type of) system

Scientific background, rationale: What is known about the (type of) system that is the object of the study (be sure to discuss the use of similar systems for other conditions/diagnoses, if appropiate), motivation for the study, i.e. what are the reasons for and what is the context for this specific study, from which stakeholder viewpoint is the study performed, potential impact of findings [2]. Briefly justify the choice of the comparator.

subitem not at all important

1 ○

2 ○

3 ○

4 ○

5 ○

essential

Does your paper address subitem 2a-ii? *

Copy and paste relevant sections from the manuscript (include quotes in quotation marks "like this" to indicate direct quotes from your manuscript), or elaborate on this item by providing additional information not in the ms, or briefly explain why the item is not applicable/relevant for your study

Digital solutions may have the potential to improve outreach and provide easy, relatively low-cost access to pain self-management interventions. Existing digital pain self-management interventions have for example shown promising results in meeting unfulfilled needs, supporting psychological well-being and improving health-related quality of life. However, a number of challenges with existing digital pain management programs exist, such as lack of theoretical foundation, such as evidence-based psychosocial interventional aspects, and lack of user-centeredness, with limited involvement of end-users (ie, people with chronic pain) and healthcare providers in the development process. In addition rigorous efficacy testing establishing efficacy appears scarce in existing digital pain self-management interventions.

2b) In INTRODUCTION: Specific objectives or hypotheses

Du endrer svaret ditt. Hvis du deler denne nettadressen, kan andre også endre svaret ditt.

FYLL UT ET NYTT SVAR

Does your paper address CONSORT subitem 2b? *

Copy and paste relevant sections from the manuscript (include quotes in quotation marks "like this" to indicate direct quotes from your manuscript), or elaborate on this item by providing additional information not in the ms, or briefly explain why the item is not applicable/relevant for your study

"The current study aimed to examine short-term (ie, three months) efficacy findings from an RCT testing the digital pain self-management intervention program EPIO. It was hypothesized that participants receiving EPIO, compared to participants in a care-as-usual control group, would experience significant improvements in primary (ie, pain interference) and secondary (ie, depression, anxiety, self-regulatory fatigue, health-related quality of life (HRQoL), pain catastrophizing, and pain acceptance) outcomes after 3 months' access to the EPIO program. System use, usefulness, and ease of use were examined on an exploratory basis".

METHODS

3a) Description of trial design (such as parallel, factorial) including allocation ratio

Does your paper address CONSORT subitem 3a? *

Copy and paste relevant sections from the manuscript (include quotes in quotation marks "like this" to indicate direct quotes from your manuscript), or elaborate on this item by providing additional information not in the ms, or briefly explain why the item is not applicable/relevant for your study

"A two-armed RCT was employed with participants randomly assigned to; (a) the digital pain self-management intervention program EPIO, or (b) a care-as-usual control group".

3b) Important changes to methods after trial commencement (such as eligibility criteria), with reasons

Du endrer svaret ditt. Hvis du deler denne nettadressen, kan andre også endre svaret ditt.

FYLL UT ET NYTT SVAR

Does your paper address CONSORT subitem 3b? *

Copy and paste relevant sections from the manuscript (include quotes in quotation marks "like this" to indicate direct quotes from your manuscript), or elaborate on this item by providing additional information not in the ms, or briefly explain why the item is not applicable/relevant for your study

There were no changes to methods after trial commencement

3b-i) Bug fixes, Downtimes, Content Changes

Bug fixes, Downtimes, Content Changes: ehealth systems are often dynamic systems. A description of changes to methods therefore also includes important changes made on the intervention or comparator during the trial (e.g., major bug fixes or changes in the functionality or content) (5-iii) and other "unexpected events" that may have influenced study design such as staff changes, system failures/downtimes, etc. [2].

subitem not at all important

1 ○

2 ○

3 ○

4 ○

5 ○

essential

Does your paper address subitem 3b-i?

Copy and paste relevant sections from the manuscript (include quotes in quotation marks "like this" to indicate direct quotes from your manuscript), or elaborate on this item by providing additional information not in the ms, or briefly explain why the item is not applicable/relevant for your study

Svaret ditt

Du endrer svaret ditt. Hvis du deler denne nettadressen, kan andre også endre svaret ditt.

FYLL UT ET NYTT SVAR

Does your paper address CONSORT subitem 4a? *

Copy and paste relevant sections from the manuscript (include quotes in quotation marks "like this" to indicate direct quotes from your manuscript), or elaborate on this item by providing additional information not in the ms, or briefly explain why the item is not applicable/relevant for your study

"Eligibility criteria included: (1) living with chronic pain in general (ie, not pain condition-specific); (2) pain duration ≥ 3 months (ie, self-reported); (3) age ≥ 18 years; (4) access to a smartphone or tablet; (5) being able to understand oral and written Norwegian; and (6) being able to attend an in-person introduction session either at a nearby health care facility or through using a secure video link (ie, due to pandemic restrictions as of spring 2020)".

4a-i) Computer / Internet literacy

Computer / Internet literacy is often an implicit "de facto" eligibility criterion - this should be explicitly clarified.

subitem not at all important

1 ○

2 ○

3 ○

4 ○

5 ○

essential

Does your paper address subitem 4a-i?

Copy and paste relevant sections from the manuscript (include quotes in quotation marks "like this" to indicate direct quotes from your manuscript), or elaborate on this item by providing additional information not in the ms, or briefly explain why the item is not applicable/relevant for your study

Svaret ditt

Du endrer svaret ditt. Hvis du deler denne nettadressen, kan andre også endre svaret ditt.

FYLL UT ET NYTT SVAR

4a-ii) Open vs. closed, web-based vs. face-to-face assessments:

Open vs. closed, web-based vs. face-to-face assessments: Mention how participants were recruited (online vs. offline), e.g., from an open access website or from a clinic, and clarify if this was a purely web-based trial, or there were face-to-face components (as part of the intervention or for assessment), i.e., to what degree got the study team to know the participant. In online-only trials, clarify if participants were quasi-anonymous and whether having multiple identities was possible or whether technical or logistical measures (e.g., cookies, email confirmation, phone calls) were used to detect/prevent these.

subitem not at all important

1 ○

2 ○

3 ○

4 ◉

5 ○

essential

Opphev valget

Does your paper address subitem 4a-ii? *

Copy and paste relevant sections from the manuscript (include quotes in quotation marks "like this" to indicate direct quotes from your manuscript), or elaborate on this item by providing additional information not in the ms, or briefly explain why the item is not applicable/relevant for your study

"Patients living with chronic pain were informed about the EPIO study verbally by collaborating partners, or through flyers, at various health care sites. If interested, the patient's contact information was forwarded to the project team, who then provided additional information about study participation to those interested. Patients could also contact study staff directly through a study phone number or website. Study information was also published through social media (eg, Facebook), and these posts were frequently re-published by individuals or patient organizations".

Du endrer svaret ditt. Hvis du deler denne nettadressen, kan andre også endre svaret ditt.

FYLL UT ET NYTT SVAR

4a-iii) Information giving during recruitment

Information given during recruitment. Specify how participants were briefed for recruitment and in the informed consent procedures (e.g., publish the informed consent documentation as appendix, see also item X26), as this information may have an effect on user self-selection, user expectation and may also bias results.

subitem not at all important

1 ○

2 ○

3 ○

4 ○

5 ○

essential

Does your paper address subitem 4a-iii?

Copy and paste relevant sections from the manuscript (include quotes in quotation marks "like this" to indicate direct quotes from your manuscript), or elaborate on this item by providing additional information not in the ms, or briefly explain why the item is not applicable/relevant for your study

Svaret ditt

4b) Settings and locations where the data were collected

Does your paper address CONSORT subitem 4b? *

Copy and paste relevant sections from the manuscript (include quotes in quotation marks "like this" to indicate direct quotes from your manuscript), or elaborate on this item by providing additional information not in the ms, or briefly explain why the item is not applicable/relevant for your study

Data were collected trough a secure research server at Services for Sensitive Data (TSD)

Du endrer svaret ditt. Hvis du deler denne nettadressen, kan andre også endre svaret ditt.

FYLL UT ET NYTT SVAR

4b-i) Report if outcomes were (self-)assessed through online questionnaires

Clearly report if outcomes were (self-)assessed through online questionnaires (as common in web-based trials) or otherwise.

subitem not at all important

1 ○

2 ○

3 ○

4 ◉

5 ○

essential

Opphev valget

Does your paper address subitem 4b-i? *

Copy and paste relevant sections from the manuscript (include quotes in quotation marks "like this" to indicate direct quotes from your manuscript), or elaborate on this item by providing additional information not in the ms, or briefly explain why the item is not applicable/relevant for your study

Outcome measures were self-asses through online questionnaires

Du endrer svaret ditt. Hvis du deler denne nettadressen, kan andre også endre svaret ditt.

FYLL UT ET NYTT SVAR

4b-ii) Report how institutional affiliations are displayed

Report how institutional affiliations are displayed to potential participants [on ehealth media], as affiliations with prestigious hospitals or universities may affect volunteer rates, use, and reactions with regards to an intervention.(Not a required item – describe only if this may bias results)

subitem not at all important

1 ○

2 ○

3 ○

4 ○

5 ○

essential

Does your paper address subitem 4b-ii?

Copy and paste relevant sections from the manuscript (include quotes in quotation marks "like this" to indicate direct quotes from your manuscript), or elaborate on this item by providing additional information not in the ms, or briefly explain why the item is not applicable/relevant for your study

Svaret ditt

5) The interventions for each group with sufficient details to allow replication, including how and when they were actually administered

Du endrer svaret ditt. Hvis du deler denne nettadressen, kan andre også endre svaret ditt.

FYLL UT ET NYTT SVAR

5-i) Mention names, credential, affiliations of the developers, sponsors, and owners

Mention names, credential, affiliations of the developers, sponsors, and owners [6] (if authors/evaluators are owners or developer of the software, this needs to be declared in a "Conflict of interest" section or mentioned elsewhere in the manuscript).

subitem not at all important

1 ○

2 ○

3 ○

4 ○

5 ○

essential

Does your paper address subitem 5-i?

Copy and paste relevant sections from the manuscript (include quotes in quotation marks "like this" to indicate direct quotes from your manuscript), or elaborate on this item by providing additional information not in the ms, or briefly explain why the item is not applicable/relevant for your study

Svaret ditt

Du endrer svaret ditt. Hvis du deler denne nettadressen, kan andre også endre svaret ditt.

FYLL UT ET NYTT SVAR

5-ii) Describe the history/development process

Describe the history/development process of the application and previous formative evaluations (e.g., focus groups, usability testing), as these will have an impact on adoption/use rates and help with interpreting results.

subitem not at all important

1 ○

2 ○

3 ○

4 ○

5 ○

essential

Does your paper address subitem 5-ii?

Copy and paste relevant sections from the manuscript (include quotes in quotation marks "like this" to indicate direct quotes from your manuscript), or elaborate on this item by providing additional information not in the ms, or briefly explain why the item is not applicable/relevant for your study

Svaret ditt

Du endrer svaret ditt. Hvis du deler denne nettadressen, kan andre også endre svaret ditt.

FYLL UT ET NYTT SVAR

5-iii) Revisions and updating

Revisions and updating. Clearly mention the date and/or version number of the application/intervention (and comparator, if applicable) evaluated, or describe whether the intervention underwent major changes during the evaluation process, or whether the development and/or content was "frozen" during the trial. Describe dynamic components such as news feeds or changing content which may have an impact on the replicability of the intervention (for unexpected events see item 3b).

subitem not at all important

1 ○

2 ○

3 ○

4 ○

5 ○

essential

Does your paper address subitem 5-iii?

Copy and paste relevant sections from the manuscript (include quotes in quotation marks "like this" to indicate direct quotes from your manuscript), or elaborate on this item by providing additional information not in the ms, or briefly explain why the item is not applicable/relevant for your study

Svaret ditt

Du endrer svaret ditt. Hvis du deler denne nettadressen, kan andre også endre svaret ditt.

FYLL UT ET NYTT SVAR

5-iv) Quality assurance methods

Provide information on quality assurance methods to ensure accuracy and quality of information provided [1], if applicable.

subitem not at all important

1 ○

2 ○

3 ○

4 ○

5 ○

essential

Does your paper address subitem 5-iv?

Copy and paste relevant sections from the manuscript (include quotes in quotation marks "like this" to indicate direct quotes from your manuscript), or elaborate on this item by providing additional information not in the ms, or briefly explain why the item is not applicable/relevant for your study

Svaret ditt

Du endrer svaret ditt. Hvis du deler denne nettadressen, kan andre også endre svaret ditt.

FYLL UT ET NYTT SVAR

5-v) Ensure replicability by publishing the source code, and/or providing screenshots/screen-capture video, and/or providing flowcharts of the algorithms used

Ensure replicability by publishing the source code, and/or providing screenshots/screen-capture video, and/or providing flowcharts of the algorithms used. Replicability (i.e., other researchers should in principle be able to replicate the study) is a hallmark of scientific reporting.

subitem not at all important

1 ○

2 ○

3 ○

4 ○

5 ○

essential

Does your paper address subitem 5-v?

Copy and paste relevant sections from the manuscript (include quotes in quotation marks "like this" to indicate direct quotes from your manuscript), or elaborate on this item by providing additional information not in the ms, or briefly explain why the item is not applicable/relevant for your study

Svaret ditt

Du endrer svaret ditt. Hvis du deler denne nettadressen, kan andre også endre svaret ditt.

FYLL UT ET NYTT SVAR

5-vi) Digital preservation

Digital preservation: Provide the URL of the application, but as the intervention is likely to change or disappear over the course of the years; also make sure the intervention is archived (Internet Archive, webcitation.org, and/or publishing the source code or screenshots/videos alongside the article). As pages behind login screens cannot be archived, consider creating demo pages which are accessible without login.

subitem not at all important

1 ○

2 ○

3 ○

4 ○

5 ○

essential

Does your paper address subitem 5-vi?

Copy and paste relevant sections from the manuscript (include quotes in quotation marks "like this" to indicate direct quotes from your manuscript), or elaborate on this item by providing additional information not in the ms, or briefly explain why the item is not applicable/relevant for your study

Svaret ditt

Du endrer svaret ditt. Hvis du deler denne nettadressen, kan andre også endre svaret ditt.

FYLL UT ET NYTT SVAR

5-vii) Access

Access: Describe how participants accessed the application, in what setting/context, if they had to pay (or were paid) or not, whether they had to be a member of specific group. If known, describe how participants obtained “access to the platform and Internet” [1]. To ensure access for editors/reviewers/readers, consider to provide a “backdoor” login account or demo mode for reviewers/readers to explore the application (also important for archiving purposes, see vi).

subitem not at all important

1 ○

2 ○

3 ○

4 ○

5 ○

essential

Does your paper address subitem 5-vii? *

Copy and paste relevant sections from the manuscript (include quotes in quotation marks "like this" to indicate direct quotes from your manuscript), or elaborate on this item by providing additional information not in the ms, or briefly explain why the item is not applicable/relevant for your study

The intervention group participated in a face-to-face introduction session where they received help downloading the EPIO application (app) from App Store or Google Play, and guidance in how to use the program. The introduction session was initially planned as being in-person, but was also provided digitally as of spring 2020 due to the occurring COVID-19 pandemic.

Du endrer svaret ditt. Hvis du deler denne nettadressen, kan andre også endre svaret ditt.

FYLL UT ET NYTT SVAR

5-viii) Mode of delivery, features/functionalities/components of the intervention and comparator, and the theoretical framework

Describe mode of delivery, features/functionalities/components of the intervention and comparator, and the theoretical framework [6] used to design them (instructional strategy [1], behaviour change techniques, persuasive features, etc., see e.g., [7, 8] for terminology). This includes an in-depth description of the content (including where it is coming from and who developed it) [1]," whether [and how] it is tailored to individual circumstances and allows users to track their progress and receive feedback" [6]. This also includes a description of communication delivery channels and – if computer-mediated communication is a component – whether communication was synchronous or asynchronous [6]. It also includes information on presentation strategies [1], including page design principles, average amount of text on pages, presence of hyperlinks to other resources, etc. [1].

subitem not at all important

1 ○

2 ○

3 ○

4 ◉

5 ○

essential

Opphev valget

Du endrer svaret ditt. Hvis du deler denne nettadressen, kan andre også endre svaret ditt.

FYLL UT ET NYTT SVAR

Does your paper address subitem 5-viii? *

Copy and paste relevant sections from the manuscript (include quotes in quotation marks "like this" to indicate direct quotes from your manuscript), or elaborate on this item by providing additional information not in the ms, or briefly explain why the item is not applicable/relevant for your study

The app-based EPIO program was delivered in a blended-care model consisting of. 1) introduction session, 2) access to the app-based program, and 3) follow-up phone calls.

"The app-based EPIO program consists of 9 CBT-based modules with aspects of ACT, each combining educational information (eg, thought challenges, coping strategies, values and activity pacing) with practical and related exercises (eg, diaphragmatic breathing, progressive muscle relaxation, visualization and mindfulness) for people living with chronic pain. The 9 modules include: (1) information about pain, (2) balance, (3) thoughts and feelings, (4) stress and coping, (5) what is important to me (ie, values), (6) behaviors and lifestyle, (7) communication, relations and social support, (8) coping during difficult times, and (9) summary and the road ahead. Through the EPIO program, progression is supported and guided by the EPIOS bird, an avatar "accompanying" the participants and their program progression. Participants are encouraged to practice content frequently to familiarize themselves with the psychoeducational content and exercises, and a 3-day "practice mode" delay has therefore been incorporated between modules (ie, from completing 1 module until the next module becomes available). The first 5 EPIO modules are sequential due to educational structure, while the participants can choose the order of modules 6 to 8 to allow for individualization. As participants proceed through the program, they can also create a list of favorites by highlighting exercises and topics, and they can opt to receive reminders based on their own needs and preferences. EPIO also allows the user to choose between reading and listening, or do both, and there is an option to use the program while offline. More detailed information about the design and development of the EPIO intervention program has been presented elsewhere"

Du endrer svaret ditt. Hvis du deler denne nettadressen, kan andre også endre svaret ditt.

FYLL UT ET NYTT SVAR

5-ix) Describe use parameters

Describe use parameters (e.g., intended “doses” and optimal timing for use). Clarify what instructions or recommendations were given to the user, e.g., regarding timing, frequency, heaviness of use, if any, or was the intervention used ad libitum.

subitem not at all important

1 ○

2 ○

3 ○

4 ○

5 ○

essential

Does your paper address subitem 5-ix?

Copy and paste relevant sections from the manuscript (include quotes in quotation marks "like this" to indicate direct quotes from your manuscript), or elaborate on this item by providing additional information not in the ms, or briefly explain why the item is not applicable/relevant for your study

Svaret ditt

Du endrer svaret ditt. Hvis du deler denne nettadressen, kan andre også endre svaret ditt.

FYLL UT ET NYTT SVAR

5-x) Clarify the level of human involvement

Clarify the level of human involvement (care providers or health professionals, also technical assistance) in the e-intervention or as co-intervention (detail number and expertise of professionals involved, if any, as well as “type of assistance offered, the timing and frequency of the support, how it is initiated, and the medium by which the assistance is delivered”. It may be necessary to distinguish between the level of human involvement required for the trial, and the level of human involvement required for a routine application outside of a RCT setting (discuss under item 21 – generalizability).

subitem not at all important

1 ○

2 ○

3 ○

4 ○

5 ○

essential

Does your paper address subitem 5-x?

Copy and paste relevant sections from the manuscript (include quotes in quotation marks "like this" to indicate direct quotes from your manuscript), or elaborate on this item by providing additional information not in the ms, or briefly explain why the item is not applicable/relevant for your study

Svaret ditt

Du endrer svaret ditt. Hvis du deler denne nettadressen, kan andre også endre svaret ditt.

FYLL UT ET NYTT SVAR

5-xi) Report any prompts/reminders used

Report any prompts/reminders used: Clarify if there were prompts (letters, emails, phone calls, SMS) to use the application, what triggered them, frequency etc. It may be necessary to distinguish between the level of prompts/reminders required for the trial, and the level of prompts/reminders for a routine application outside of a RCT setting (discuss under item 21 – generalizability).

subitem not at all important

1 ○

2 ○

3 ◉

4 ○

5 ○

essential

Opphev valget

Does your paper address subitem 5-xi? *

Copy and paste relevant sections from the manuscript (include quotes in quotation marks "like this" to indicate direct quotes from your manuscript), or elaborate on this item by providing additional information not in the ms, or briefly explain why the item is not applicable/relevant for your study

"The intervention group received follow-up phone calls at approximately 3 and 7 weeks to see how they were doing. The project team could also be contacted through the project study phone for questions or technical assistance".

Du endrer svaret ditt. Hvis du deler denne nettadressen, kan andre også endre svaret ditt.

FYLL UT ET NYTT SVAR

5-xii) Describe any co-interventions (incl. training/support)

Describe any co-interventions (incl. training/support): Clearly state any interventions that are provided in addition to the targeted eHealth intervention, as ehealth intervention may not be designed as stand-alone intervention. This includes training sessions and support [1]. It may be necessary to distinguish between the level of training required for the trial, and the level of training for a routine application outside of a RCT setting (discuss under item 21 – generalizability.

subitem not at all important

1 ○

2 ○

3 ◉

4 ○

5 ○

essential

Opphev valget

Does your paper address subitem 5-xii? *

Copy and paste relevant sections from the manuscript (include quotes in quotation marks "like this" to indicate direct quotes from your manuscript), or elaborate on this item by providing additional information not in the ms, or briefly explain why the item is not applicable/relevant for your study

There were no co-intervention in addition to the targeted eHealth intervention

6a) Completely defined pre-specified primary and secondary outcome measures, including how and when they were assessed

Du endrer svaret ditt. Hvis du deler denne nettadressen, kan andre også endre svaret ditt.

FYLL UT ET NYTT SVAR

Does your paper address CONSORT subitem 6a? *

Copy and paste relevant sections from the manuscript (include quotes in quotation marks "like this" to indicate direct quotes from your manuscript), or elaborate on this item by providing additional information not in the ms, or briefly explain why the item is not applicable/relevant for your study

Outcome measures were collected at baseline (ie, before randomization) and at 3 months after the introduction session.

Outcome measures included pain interference (BPI; primary outcome measure), anxiety and depression (HADS), self-regulatory fatigue (SRF-18), health-related quality of life (SF-36/RAND-36), pain catastrophizing (PCS), and pain acceptance (CPAQ).

6a-i) Online questionnaires: describe if they were validated for online use and apply CHERRIES items to describe how the questionnaires were designed/deployed

If outcomes were obtained through online questionnaires, describe if they were validated for online use and apply CHERRIES items to describe how the questionnaires were designed/deployed [9].

subitem not at all important

1 ○

2 ○

3 ○

4 ○

5 ○

essential

Does your paper address subitem 6a-i?

Copy and paste relevant sections from manuscript text

Svaret ditt

Du endrer svaret ditt. Hvis du deler denne nettadressen, kan andre også endre svaret ditt.

FYLL UT ET NYTT SVAR

6a-ii) Describe whether and how “use” (including intensity of use/dosage) was defined/measured/monitored

Describe whether and how “use” (including intensity of use/dosage) was defined/measured/monitored (logins, logfile analysis, etc.). Use/adoption metrics are important process outcomes that should be reported in any ehealth trial.

subitem not at all important

1 ○

2 ○

3 ○

4 ○

5 ○

essential

Does your paper address subitem 6a-ii?

Copy and paste relevant sections from manuscript text

Svaret ditt

Du endrer svaret ditt. Hvis du deler denne nettadressen, kan andre også endre svaret ditt.

FYLL UT ET NYTT SVAR

6a-iii) Describe whether, how, and when qualitative feedback from participants was obtained

Describe whether, how, and when qualitative feedback from participants was obtained (e.g., through emails, feedback forms, interviews, focus groups).

subitem not at all important

1 ○

2 ○

3 ○

4 ○

5 ○

essential

Does your paper address subitem 6a-iii?

Copy and paste relevant sections from manuscript text

Svaret ditt

6b) Any changes to trial outcomes after the trial commenced, with reasons

Does your paper address CONSORT subitem 6b? *

Copy and paste relevant sections from the manuscript (include quotes in quotation marks "like this" to indicate direct quotes from your manuscript), or elaborate on this item by providing additional information not in the ms, or briefly explain why the item is not applicable/relevant for your study

There were no changes to trial outcomes after the trial commenced

Du endrer svaret ditt. Hvis du deler denne nettadressen, kan andre også endre svaret ditt.

FYLL UT ET NYTT SVAR

7a) How sample size was determined

NPT: When applicable, details of whether and how the clustering by care provides or centers was addressed

7a-i) Describe whether and how expected attrition was taken into account when calculating the sample size

Describe whether and how expected attrition was taken into account when calculating the sample size.

subitem not at all important

1 ○

2 ○

3 ○

4 ◉

5 ○

essential

Opphev valget

Does your paper address subitem 7a-i?

Copy and paste relevant sections from manuscript title (include quotes in quotation marks "like this" to indicate direct quotes from your manuscript), or elaborate on this item by providing additional information not in the ms, or briefly explain why the item is not applicable/relevant for your study

"eHealth interventions for comparable samples have shown a Cohen's d effect size of 0.30-0.40 on primary outcome (ie, pain interference). Based on these findings, a sample size for intervention effect on pain interference was calculated to allow detection of d=0.4, with an alpha of 0.05 and 80% power (based on a 2 tailed t-test). This included a total of 200 participants. To account for attrition and allow for adequate power in secondary outcome analyses, a total of 266 participants were included".

Du endrer svaret ditt. Hvis du deler denne nettadressen, kan andre også endre svaret ditt.

FYLL UT ET NYTT SVAR

Does your paper address CONSORT subitem 7b? *

Copy and paste relevant sections from the manuscript (include quotes in quotation marks "like this" to indicate direct quotes from your manuscript), or elaborate on this item by providing additional information not in the ms, or briefly explain why the item is not applicable/relevant for your study

There were no interim analyses and stopping guidelines

8a) Method used to generate the random allocation sequence

NPT: When applicable, how care providers were allocated to each trial group

Does your paper address CONSORT subitem 8a? *

Copy and paste relevant sections from the manuscript (include quotes in quotation marks "like this" to indicate direct quotes from your manuscript), or elaborate on this item by providing additional information not in the ms, or briefly explain why the item is not applicable/relevant for your study

Randomization, either to the EPIO intervention or the care-as-usual control group, was computerized and stratified by gender, with study arms 1:1 and block size 20.

8b) Type of randomisation; details of any restriction (such as blocking and block size)

Does your paper address CONSORT subitem 8b? *

Copy and paste relevant sections from the manuscript (include quotes in quotation marks "like this" to indicate direct quotes from your manuscript), or elaborate on this item by providing additional information not in the ms, or briefly explain why the item is not applicable/relevant for your study

Randomization, either to the EPIO intervention or the care-as-usual control group, was computerized and stratified by gender, with study arms 1:1 and block size 20.

Du endrer svaret ditt. Hvis du deler denne nettadressen, kan andre også endre svaret ditt.

FYLL UT ET NYTT SVAR

9) Mechanism used to implement the random allocation sequence (such as sequentially numbered containers), describing any steps taken to conceal the sequence until interventions were assigned

Does your paper address CONSORT subitem 9? *

Copy and paste relevant sections from the manuscript (include quotes in quotation marks "like this" to indicate direct quotes from your manuscript), or elaborate on this item by providing additional information not in the ms, or briefly explain why the item is not applicable/relevant for your study

Randomization, either to the EPIO intervention or the care-as-usual control group, was computerized and stratified by gender, with study arms 1:1 and block size 20.

10) Who generated the random allocation sequence, who enrolled participants, and who assigned participants to interventions

Does your paper address CONSORT subitem 10? *

Copy and paste relevant sections from the manuscript (include quotes in quotation marks "like this" to indicate direct quotes from your manuscript), or elaborate on this item by providing additional information not in the ms, or briefly explain why the item is not applicable/relevant for your study

Randomization, either to the EPIO intervention or the care-as-usual control group, was computerized and stratified by gender, with study arms 1:1 and block size 20.

11a) If done, who was blinded after assignment to interventions (for example, participants, care providers, those assessing outcomes) and how
NPT: Whether or not administering co-interventions were blinded to group assignment

Du endrer svaret ditt. Hvis du deler denne nettadressen, kan andre også endre svaret ditt.

FYLL UT ET NYTT SVAR

11a-i) Specify who was blinded, and who wasn't

Specify who was blinded, and who wasn't. Usually, in web-based trials it is not possible to blind the participants [1, 3] (this should be clearly acknowledged), but it may be possible to blind outcome assessors, those doing data analysis or those administering co-interventions (if any).

subitem not at all important

1 ○

2 ○

3 ○

4 ○

5 ○

essential

Does your paper address subitem 11a-i? *

Copy and paste relevant sections from the manuscript (include quotes in quotation marks "like this" to indicate direct quotes from your manuscript), or elaborate on this item by providing additional information not in the ms, or briefly explain why the item is not applicable/relevant for your study

Not applicable to this trial

Du endrer svaret ditt. Hvis du deler denne nettadressen, kan andre også endre svaret ditt.

FYLL UT ET NYTT SVAR

11a-ii) Discuss e.g., whether participants knew which intervention was the "intervention of interest" and which one was the "comparator"

Informed consent procedures (4a-ii) can create biases and certain expectations - discuss e.g., whether participants knew which intervention was the "intervention of interest" and which one was the "comparator".

subitem not at all important

1 ○

2 ○

3 ○

4 ○

5 ○

essential

Does your paper address subitem 11a-ii?

Copy and paste relevant sections from the manuscript (include quotes in quotation marks "like this" to indicate direct quotes from your manuscript), or elaborate on this item by providing additional information not in the ms, or briefly explain why the item is not applicable/relevant for your study

Not applicable to this trial

11b) If relevant, description of the similarity of interventions

(this item is usually not relevant for ehealth trials as it refers to similarity of a placebo or sham intervention to a active medication/intervention)

Du endrer svaret ditt. Hvis du deler denne nettadressen, kan andre også endre svaret ditt.

FYLL UT ET NYTT SVAR

Does your paper address CONSORT subitem 11b? *

Copy and paste relevant sections from the manuscript (include quotes in quotation marks "like this" to indicate direct quotes from your manuscript), or elaborate on this item by providing additional information not in the ms, or briefly explain why the item is not applicable/relevant for your study

Not applicable to this trial

12a) Statistical methods used to compare groups for primary and secondary outcomes

NPT: When applicable, details of whether and how the clustering by care providers or centers was addressed

Does your paper address CONSORT subitem 12a? *

Copy and paste relevant sections from the manuscript (include quotes in quotation marks "like this" to indicate direct quotes from your manuscript), or elaborate on this item by providing additional information not in the ms, or briefly explain why the item is not applicable/relevant for your study

"Change scores, defined as a difference between scores at baseline and at 3 months, were calculated for pain interference, anxiety, depression, self-regulatory fatigue, HRQoL, pain catastrophizing and pain acceptance, and used as dependent variables in linear regression models".

Du endrer svaret ditt. Hvis du deler denne nettadressen, kan andre også endre svaret ditt.

FYLL UT ET NYTT SVAR

12a-i) Imputation techniques to deal with attrition / missing values

Imputation techniques to deal with attrition / missing values: Not all participants will use the intervention/comparator as intended and attrition is typically high in ehealth trials. Specify how participants who did not use the application or dropped out from the trial were treated in the statistical analysis (a complete case analysis is strongly discouraged, and simple imputation techniques such as LOCF may also be problematic [4]).

subitem not at all important

1 ○

2 ○

3 ○

4 ○

5 ○

essential

Does your paper address subitem 12a-i? *

Copy and paste relevant sections from the manuscript (include quotes in quotation marks "like this" to indicate direct quotes from your manuscript), or elaborate on this item by providing additional information not in the ms, or briefly explain why the item is not applicable/relevant for your study

Intention to treat analysis

12b) Methods for additional analyses, such as subgroup analyses and adjusted analyses

Du endrer svaret ditt. Hvis du deler denne nettadressen, kan andre også endre svaret ditt.

FYLL UT ET NYTT SVAR

Does your paper address CONSORT subitem 12b? *

Copy and paste relevant sections from the manuscript (include quotes in quotation marks "like this" to indicate direct quotes from your manuscript), or elaborate on this item by providing additional information not in the ms, or briefly explain why the item is not applicable/relevant for your study

"As statistically significant differences were observed between the intervention and the care-as-usual control group for age and years living with pain, these variables were included in the intention-to-treat analysis as possible confounders".

X26) REB/IRB Approval and Ethical Considerations [recommended as subheading under "Methods"] (not a CONSORT item)

X26-i) Comment on ethics committee approval

subitem not at all important

1 ○

2 ○

3 ○

4 ○

5 ○

essential

Does your paper address subitem X26-i?

Copy and paste relevant sections from the manuscript (include quotes in quotation marks "like this" to indicate direct quotes from your manuscript), or elaborate on this item by providing additional information not in the ms, or briefly explain why the item is not applicable/relevant for your study

Svaret ditt

Du endrer svaret ditt. Hvis du deler denne nettadressen, kan andre også endre svaret ditt.

FYLL UT ET NYTT SVAR

x26-ii) Outline informed consent procedures

Outline informed consent procedures e.g., if consent was obtained offline or online (how? Checkbox, etc.?), and what information was provided (see 4a-ii). See [6] for some items to be included in informed consent documents.

subitem not at all important

1 ○

2 ○

3 ○

4 ○

5 ○

essential

Does your paper address subitem X26-ii?

Copy and paste relevant sections from the manuscript (include quotes in quotation marks "like this" to indicate direct quotes from your manuscript), or elaborate on this item by providing additional information not in the ms, or briefly explain why the item is not applicable/relevant for your study

Svaret ditt

Du endrer svaret ditt. Hvis du deler denne nettadressen, kan andre også endre svaret ditt.

FYLL UT ET NYTT SVAR

X26-iii) Safety and security procedures

Safety and security procedures, incl. privacy considerations, and any steps taken to reduce the likelihood or detection of harm (e.g., education and training, availability of a hotline)

subitem not at all important

1 ○

2 ○

3 ○

4 ○

5 ○

essential

Does your paper address subitem X26-iii?

Copy and paste relevant sections from the manuscript (include quotes in quotation marks "like this" to indicate direct quotes from your manuscript), or elaborate on this item by providing additional information not in the ms, or briefly explain why the item is not applicable/relevant for your study

Svaret ditt

RESULTS

13a) For each group, the numbers of participants who were randomly assigned, received intended treatment, and were analysed for the primary outcome

NPT: The number of care providers or centers performing the intervention in each group and the number of patients treated by each care provider in each center

Du endrer svaret ditt. Hvis du deler denne nettadressen, kan andre også endre svaret ditt.

FYLL UT ET NYTT SVAR

Does your paper address CONSORT subitem 13a? *

Copy and paste relevant sections from the manuscript (include quotes in quotation marks "like this" to indicate direct quotes from your manuscript), or elaborate on this item by providing additional information not in the ms, or briefly explain why the item is not applicable/relevant for your study

Patients (N=266) were randomly assigned to the EPIO intervention (n=132) or a care-as-usual control group (n=134). Seven participants allocated to the intervention group withdrew from the study or did not respond before the introduction session, and were therefore excluded from the study. Final study sample was hence 259, with 125 participants in the intervention group and 134 in the control group. A total of 229 participants completed the outcome measures at 3-month follow-up (intervention group n=109; control group n=120) and were analyzed for the primary outcome.

13b) For each group, losses and exclusions after randomisation, together with reasons

Does your paper address CONSORT subitem 13b? (NOTE: Preferably, this is shown in a CONSORT flow diagram) *

Copy and paste relevant sections from the manuscript (include quotes in quotation marks "like this" to indicate direct quotes from your manuscript), or elaborate on this item by providing additional information not in the ms, or briefly explain why the item is not applicable/relevant for your study

Seven participants allocated to the intervention group withdrew from the study or did not respond before the introduction session, and were therefore excluded from the study. Final study sample was hence 259, with 125 participants in the intervention group and 134 in the control group.

Du endrer svaret ditt. Hvis du deler denne nettadressen, kan andre også endre svaret ditt.

FYLL UT ET NYTT SVAR

13b-i) Attrition diagram

Strongly recommended: An attrition diagram (e.g., proportion of participants still logging in or using the intervention/comparator in each group plotted over time, similar to a survival curve) or other figures or tables demonstrating usage/dose/engagement.

subitem not at all important

1 ○

2 ○

3 ○

4 ○

5 ○

essential

Does your paper address subitem 13b-i?

Copy and paste relevant sections from the manuscript or cite the figure number if applicable (include quotes in quotation marks "like this" to indicate direct quotes from your manuscript), or elaborate on this item by providing additional information not in the ms, or briefly explain why the item is not applicable/relevant for your study

Svaret ditt

14a) Dates defining the periods of recruitment and follow-up

Does your paper address CONSORT subitem 14a? *

Copy and paste relevant sections from the manuscript (include quotes in quotation marks "like this" to indicate direct quotes from your manuscript), or elaborate on this item by providing additional information not in the ms, or briefly explain why the item is not applicable/relevant for your study

Participants were people living with chronic pain, recruited November 2019 - February 2021, and follow-up through 2022.

Du endrer svaret ditt. Hvis du deler denne nettadressen, kan andre også endre svaret ditt.

FYLL UT ET NYTT SVAR

14a-i) Indicate if critical “secular events” fell into the study period

Indicate if critical “secular events” fell into the study period, e.g., significant changes in Internet resources available or “changes in computer hardware or Internet delivery resources”

subitem not at all important

1 ○

2 ○

3 ○

4 ○

5 ○

essential

Does your paper address subitem 14a-i?

Copy and paste relevant sections from the manuscript (include quotes in quotation marks "like this" to indicate direct quotes from your manuscript), or elaborate on this item by providing additional information not in the ms, or briefly explain why the item is not applicable/relevant for your study

Svaret ditt

14b) Why the trial ended or was stopped (early)

Does your paper address CONSORT subitem 14b? *

Copy and paste relevant sections from the manuscript (include quotes in quotation marks "like this" to indicate direct quotes from your manuscript), or elaborate on this item by providing additional information not in the ms, or briefly explain why the item is not applicable/relevant for your study

Not applicable to this trial

Du endrer svaret ditt. Hvis du deler denne nettadressen, kan andre også endre svaret ditt.

FYLL UT ET NYTT SVAR

15) A table showing baseline demographic and clinical characteristics for each group

NPT: When applicable, a description of care providers (case volume, qualification, expertise, etc.) and centers (volume) in each group

Does your paper address CONSORT subitem 15? *

Copy and paste relevant sections from the manuscript (include quotes in quotation marks "like this" to indicate direct quotes from your manuscript), or elaborate on this item by providing additional information not in the ms, or briefly explain why the item is not applicable/relevant for your study

"Participants (N=259) were primarily Caucasian (97%), median age 49 years (range 22-78 years) old at inclusion, and were mainly female (210/259, 81%). The majority of the participants reported being on sick leave or disability benefits (186/259, 72%) at the time of enrollment and reported having lived with pain for ten years or longer (158/259,

15-i) Report demographics associated with digital divide issues

In ehealth trials it is particularly important to report demographics associated with digital divide issues, such as age, education, gender, social-economic status, computer/Internet/ehealth literacy of the participants, if known.

subitem not at all important

- 1 ○
- 2 ○
- 3 ●
- 4 ○
- 5 ○

essential

Opphev valget

Du endrer svaret ditt. Hvis du deler denne nettadressen, kan andre også endre svaret ditt.

FYLL UT ET NYTT SVAR

Does your paper address subitem 15-i? *

Copy and paste relevant sections from the manuscript (include quotes in quotation marks "like this" to indicate direct quotes from your manuscript), or elaborate on this item by providing additional information not in the ms, or briefly explain why the item is not applicable/relevant for your study

"Participants (N=259) were primarily Caucasian (97%), median age 49 years (range 22-78 years) old at inclusion, and were mainly female (210/259, 81%). The majority of the participants reported being on sick leave or disability benefits (186/259, 72%) at the time of enrollment and reported having lived with pain for ten years or longer (158/259,

16) For each group, number of participants (denominator) included in each analysis and whether the analysis was by original assigned groups

16-i) Report multiple "denominators" and provide definitions

Report multiple "denominators" and provide definitions: Report N's (and effect sizes) "across a range of study participation [and use] thresholds" [1], e.g., N exposed, N consented, N used more than x times, N used more than y weeks, N participants "used" the intervention/comparator at specific pre-defined time points of interest (in absolute and relative numbers per group). Always clearly define "use" of the intervention.

subitem not at all important

1 ○

2 ○

3 ○

4 ○

5 ○

essential

Du endrer svaret ditt. Hvis du deler denne nettadressen, kan andre også endre svaret ditt.

FYLL UT ET NYTT SVAR

Does your paper address subitem 16-i? *

Copy and paste relevant sections from the manuscript (include quotes in quotation marks "like this" to indicate direct quotes from your manuscript), or elaborate on this item by providing additional information not in the ms, or briefly explain why the item is not applicable/relevant for your study

A total of 229 participants completed the outcome measures at 3-month follow-up (intervention group n=109; control group n=120) and were included in the analysis.

16-ii) Primary analysis should be intent-to-treat

Primary analysis should be intent-to-treat, secondary analyses could include comparing only "users", with the appropriate caveats that this is no longer a randomized sample (see 18-i).

subitem not at all important

1 ○

2 ○

3 ○

4 ○

5 ○

essential

Does your paper address subitem 16-ii?

Copy and paste relevant sections from the manuscript (include quotes in quotation marks "like this" to indicate direct quotes from your manuscript), or elaborate on this item by providing additional information not in the ms, or briefly explain why the item is not applicable/relevant for your study

Svaret ditt

17a) For each primary and secondary outcome, results for each group, and the

Du endrer svaret ditt. Hvis du deler denne nettadressen, kan andre også endre svaret ditt.

FYLL UT ET NYTT SVAR

Does your paper address CONSORT subitem 17a? *

Copy and paste relevant sections from the manuscript (include quotes in quotation marks "like this" to indicate direct quotes from your manuscript), or elaborate on this item by providing additional information not in the ms, or briefly explain why the item is not applicable/relevant for your study

"There was no statistically significant difference between the intervention and control groups for the primary outcome of pain interference on function at 3-month follow-up. There were however significant reductions in symptoms of depression (ie, MD -.90, P=.034) and self-regulatory fatigue (ie, MD -2.76, P=.008) for the EPIO intervention group compared to the care-as-usual control group. There were no statistically significant between group differences for anxiety, pain acceptance, pain catastrophizing or HRQoL at 3 months, only a trend towards statistical significance for the HRQoL subscale of Vitality (MD 4.25, P=.050) and the Chronic Pain Acceptance Item of Activity Engagement (MD .079, P=.078)".

17a-i) Presentation of process outcomes such as metrics of use and intensity of use

In addition to primary/secondary (clinical) outcomes, the presentation of process outcomes such as metrics of use and intensity of use (dose, exposure) and their operational definitions is critical. This does not only refer to metrics of attrition (13-b) (often a binary variable), but also to more continuous exposure metrics such as "average session length". These must be accompanied by a technical description how a metric like a "session" is defined (e.g., timeout after idle time) [1] (report under item 6a).

subitem not at all important

1 ○

2 ○

3 ○

4 ○

5 ○

essential

Du endrer svaret ditt. Hvis du deler denne nettadressen, kan andre også endre svaret ditt.

FYLL UT ET NYTT SVAR

Does your paper address subitem 17a-i?

Copy and paste relevant sections from the manuscript (include quotes in quotation marks "like this" to indicate direct quotes from your manuscript), or elaborate on this item by providing additional information not in the ms, or briefly explain why the item is not applicable/relevant for your study

Svaret ditt

17b) For binary outcomes, presentation of both absolute and relative effect sizes is recommended

Does your paper address CONSORT subitem 17b? *

Copy and paste relevant sections from the manuscript (include quotes in quotation marks "like this" to indicate direct quotes from your manuscript), or elaborate on this item by providing additional information not in the ms, or briefly explain why the item is not applicable/relevant for your study

Not applicable to this trial

18) Results of any other analyses performed, including subgroup analyses and adjusted analyses, distinguishing pre-specified from exploratory

Does your paper address CONSORT subitem 18? *

Copy and paste relevant sections from the manuscript (include quotes in quotation marks "like this" to indicate direct quotes from your manuscript), or elaborate on this item by providing additional information not in the ms, or briefly explain why the item is not applicable/relevant for your study

Not applicable to this trial

Du endrer svaret ditt. Hvis du deler denne nettadressen, kan andre også endre svaret ditt.

FYLL UT ET NYTT SVAR

18-i) Subgroup analysis of comparing only users

A subgroup analysis of comparing only users is not uncommon in ehealth trials, but if done, it must be stressed that this is a self-selected sample and no longer an unbiased sample from a randomized trial (see 16-iii).

subitem not at all important

1 ○

2 ○

3 ○

4 ○

5 ○

essential

Does your paper address subitem 18-i?

Copy and paste relevant sections from the manuscript (include quotes in quotation marks "like this" to indicate direct quotes from your manuscript), or elaborate on this item by providing additional information not in the ms, or briefly explain why the item is not applicable/relevant for your study

Svaret ditt

19) All important harms or unintended effects in each group

(for specific guidance see CONSORT for harms)

Does your paper address CONSORT subitem 19? *

Copy and paste relevant sections from the manuscript (include quotes in quotation marks "like this" to indicate direct quotes from your manuscript), or elaborate on this item by providing additional information not in the ms, or briefly explain why the item is not applicable/relevant for your study

There were no repoted harms or unintended effects in each group

Du endrer svaret ditt. Hvis du deler denne nettadressen, kan andre også endre svaret ditt.

FYLL UT ET NYTT SVAR

19-i) Include privacy breaches, technical problems

Include privacy breaches, technical problems. This does not only include physical “harm” to participants, but also incidents such as perceived or real privacy breaches [1], technical problems, and other unexpected/unintended incidents. “Unintended effects” also includes unintended positive effects [2].

subitem not at all important

1 ○

2 ○

3 ○

4 ○

5 ○

essential

Does your paper address subitem 19-i?

Copy and paste relevant sections from the manuscript (include quotes in quotation marks "like this" to indicate direct quotes from your manuscript), or elaborate on this item by providing additional information not in the ms, or briefly explain why the item is not applicable/relevant for your study

Svaret ditt

Du endrer svaret ditt. Hvis du deler denne nettadressen, kan andre også endre svaret ditt.

FYLL UT ET NYTT SVAR

19-ii) Include qualitative feedback from participants or observations from staff/researchers

Include qualitative feedback from participants or observations from staff/researchers, if available, on strengths and shortcomings of the application, especially if they point to unintended/unexpected effects or uses. This includes (if available) reasons for why people did or did not use the application as intended by the developers.

subitem not at all important

1 ○

2 ○

3 ○

4 ○

5 ○

essential

Does your paper address subitem 19-ii?

Copy and paste relevant sections from the manuscript (include quotes in quotation marks "like this" to indicate direct quotes from your manuscript), or elaborate on this item by providing additional information not in the ms, or briefly explain why the item is not applicable/relevant for your study

Svaret ditt

DISCUSSION

22) Interpretation consistent with results, balancing benefits and harms, and considering other relevant evidence

NPT: In addition, take into account the choice of the comparator, lack of or partial blinding, and unequal expertise of care providers or centers in each group

Du endrer svaret ditt. Hvis du deler denne nettadressen, kan andre også endre svaret ditt.

FYLL UT ET NYTT SVAR

22-i) Restate study questions and summarize the answers suggested by the data, starting with primary outcomes and process outcomes (use)

Restate study questions and summarize the answers suggested by the data, starting with primary outcomes and process outcomes (use).

subitem not at all important

1 ○

2 ○

3 ○

4 ○

5 ○

essential

Does your paper address subitem 22-i? *

Copy and paste relevant sections from the manuscript (include quotes in quotation marks "like this" to indicate direct quotes from your manuscript), or elaborate on this item by providing additional information not in the ms, or briefly explain why the item is not applicable/relevant for your study

"In this RCT, people with chronic pain having access to the digital pain self-management intervention EPIO, compared with care-as-usual controls, did not report statistically significantly different changes in the primary outcome of pain interference on function after three months, nor in the secondary outcomes of anxiety, HRQoL, pain catastrophizing or pain acceptance. Participants in the intervention group did however report significant decrease in symptoms of depression and self-regulatory fatigue, compared with care-as-usual controls, indicating that people living with chronic pain may benefit from having access to evidence-informed, user-centered digital self-management programs such as EPIO".

Du endrer svaret ditt. Hvis du deler denne nettadressen, kan andre også endre svaret ditt.

FYLL UT ET NYTT SVAR

22-ii) Highlight unanswered new questions, suggest future research

Highlight unanswered new questions, suggest future research.

subitem not at all important

1 ○

2 ○

3 ○

4 ○

5 ○

essential

Does your paper address subitem 22-ii?

Copy and paste relevant sections from the manuscript (include quotes in quotation marks "like this" to indicate direct quotes from your manuscript), or elaborate on this item by providing additional information not in the ms, or briefly explain why the item is not applicable/relevant for your study

Svaret ditt

20) Trial limitations, addressing sources of potential bias, imprecision, and, if relevant, multiplicity of analyses

Du endrer svaret ditt. Hvis du deler denne nettadressen, kan andre også endre svaret ditt.

FYLL UT ET NYTT SVAR

20-i) Typical limitations in ehealth trials

Typical limitations in ehealth trials: Participants in ehealth trials are rarely blinded. Ehealth trials often look at a multiplicity of outcomes, increasing risk for a Type I error. Discuss biases due to non-use of the intervention/usability issues, biases through informed consent procedures, unexpected events.

subitem not at all important

1 ○

2 ○

3 ○

4 ○

5 ○

essential

Does your paper address subitem 20-i? *

Copy and paste relevant sections from the manuscript (include quotes in quotation marks "like this" to indicate direct quotes from your manuscript), or elaborate on this item by providing additional information not in the ms, or briefly explain why the item is not applicable/relevant for your study

"Participants were recruited through social media and collaborating project partners, which may suggest that the study population consisted of highly motivated individuals. Whether people with chronic pain would in general be interested in or benefit from a digital self-management intervention such as EPIO is therefore not clear".

21) Generalisability (external validity, applicability) of the trial findings

NPT: External validity of the trial findings according to the intervention, comparators, patients, and care providers or centers involved in the trial

Du endrer svaret ditt. Hvis du deler denne nettadressen, kan andre også endre svaret ditt.

FYLL UT ET NYTT SVAR

21-i) Generalizability to other populations

Generalizability to other populations: In particular, discuss generalizability to a general Internet population, outside of a RCT setting, and general patient population, including applicability of the study results for other organizations

subitem not at all important

1 ○

2 ○

3 ○

4 ○

5 ○

essential

Does your paper address subitem 21-i?

Copy and paste relevant sections from the manuscript (include quotes in quotation marks "like this" to indicate direct quotes from your manuscript), or elaborate on this item by providing additional information not in the ms, or briefly explain why the item is not applicable/relevant for your study

Svaret ditt

Du endrer svaret ditt. Hvis du deler denne nettadressen, kan andre også endre svaret ditt.

FYLL UT ET NYTT SVAR

21-ii) Discuss if there were elements in the RCT that would be different in a routine application setting

Discuss if there were elements in the RCT that would be different in a routine application setting (e.g., prompts/reminders, more human involvement, training sessions or other co-interventions) and what impact the omission of these elements could have on use, adoption, or outcomes if the intervention is applied outside of a RCT setting.

subitem not at all important

1 ○

2 ○

3 ○

4 ○

5 ○

essential

Does your paper address subitem 21-ii?

Copy and paste relevant sections from the manuscript (include quotes in quotation marks "like this" to indicate direct quotes from your manuscript), or elaborate on this item by providing additional information not in the ms, or briefly explain why the item is not applicable/relevant for your study

Svaret ditt

OTHER INFORMATION

23) Registration number and name of trial registry

Du endrer svaret ditt. Hvis du deler denne nettadressen, kan andre også endre svaret ditt.

FYLL UT ET NYTT SVAR

Does your paper address CONSORT subitem 23? *

Copy and paste relevant sections from the manuscript (include quotes in quotation marks "like this" to indicate direct quotes from your manuscript), or elaborate on this item by providing additional information not in the ms, or briefly explain why the item is not applicable/relevant for your study

Study registration: the study was pre-registered at ClinicalTrials.gov NCT03705104

24) Where the full trial protocol can be accessed, if available

Does your paper address CONSORT subitem 24? *

Cite a Multimedia Appendix, other reference, or copy and paste relevant sections from the manuscript (include quotes in quotation marks "like this" to indicate direct quotes from your manuscript), or elaborate on this item by providing additional information not in the ms, or briefly explain why the item is not applicable/relevant for your study

ClinicalTrials.gov NCT03705104; https://clinicaltrials.gov/ct2/show/NCT03705104

25) Sources of funding and other support (such as supply of drugs), role of funders

Does your paper address CONSORT subitem 25? *

Copy and paste relevant sections from the manuscript (include quotes in quotation marks "like this" to indicate direct quotes from your manuscript), or elaborate on this item by providing additional information not in the ms, or briefly explain why the item is not applicable/relevant for your study

This study is funded by the Norwegian Research Council (grant #256574 awarded to Principal Investigator Lise Solberg Nes).

X27) Conflicts of Interest (not a CONSORT item)

Du endrer svaret ditt. Hvis du deler denne nettadressen, kan andre også endre svaret ditt.

FYLL UT ET NYTT SVAR

X27-i) State the relation of the study team towards the system being evaluated

In addition to the usual declaration of interests (financial or otherwise), also state the relation of the study team towards the system being evaluated, i.e., state if the authors/evaluators are distinct from or identical with the developers/sponsors of the intervention.

subitem not at all important

1 ○

2 ○

3 ○

4 ○

5 ○

essential

Does your paper address subitem X27-i?

Copy and paste relevant sections from the manuscript (include quotes in quotation marks "like this" to indicate direct quotes from your manuscript), or elaborate on this item by providing additional information not in the ms, or briefly explain why the item is not applicable/relevant for your study

Svaret ditt

About the CONSORT EHEALTH checklist

As a result of using this checklist, did you make changes in your manuscript? *

○ yes, major changes

○ yes, minor changes

◉ no

Du endrer svaret ditt. Hvis du deler denne nettadressen, kan andre også endre svaret ditt.

FYLL UT ET NYTT SVAR

What were the most important changes you made as a result of using this checklist?

Svaret ditt

How much time did you spend on going through the checklist INCLUDING making changes in your manuscript *

I used 2 hours going through the checklist

As a result of using this checklist, do you think your manuscript has improved? *

- (•) yes
- ( ) no
- ( ) Andre: ______

Would you like to become involved in the CONSORT EHEALTH group?

This would involve for example becoming involved in participating in a workshop and writing an "Explanation and Elaboration" document

- ( ) yes
- (•) no
- ( ) Andre: ______

Opphev valget

Any other comments or questions on CONSORT EHEALTH

Svaret ditt

Du endrer svaret ditt. Hvis du deler denne nettadressen, kan andre også endre svaret ditt.

FYLL UT ET NYTT SVAR

STOP - Save this form as PDF before you click submit

To generate a record that you filled in this form, we recommend to generate a PDF of this page (on a Mac, simply select "print" and then select "print as PDF") before you submit it.

When you submit your (revised) paper to JMIR, please upload the PDF as supplementary file.

Don't worry if some text in the textboxes is cut off, as we still have the complete information in our database. Thank you!

Final step: Click submit !

Click submit so we have your answers in our database!

Send

Send aldri passord via Google Skjemaer.

Dette innholdet er ikke laget eller godkjent av Google. Rapportér uriktig bruk - Vilkår for bruk - Retningslinjer for personvern

Google Skjemaer

Du endrer svaret ditt. Hvis du deler denne nettadressen, kan andre også endre svaret ditt.

FYLL UT ET NYTT SVAR

Du endrer svaret ditt. Hvis du deler denne nettadressen, kan andre også endre svaret ditt.

FYLL UT ET NYTT SVAR
