# Supplementary material for: Short-Term Findings From Testing EPIO, a Digital Self-Management Program for People Living With Chronic Pain: Randomized Controlled Trial
Source: J Med Internet Res. 2023 Aug 25;25:e47284. doi: 10.2196/47284 (PMC10492177; doi:10.2196/47284)
Supplement: Multimedia Appendix 2 [file jmir_v25i1e47284_app2.docx]

Estimates at both time points and between-group differences in the change from baseline to the 3-month follow-up.

|  | | | Intervention (n=109), mean (95% CI) | Control (n=120), mean (95% CI) | Between-group differences in change, mean difference (95% CI) | *P* value | Effect size, β |
| --- | --- | --- | --- | --- | --- | --- | --- |
| **Pain interference (BPI^a^)^b^** | | | | | −0.047 (−0.51 to 0.40) | .84 | −.014 |
|  | Baseline | | 4.84 (4.45 to 5.23) | 5.35 (4.99 to 5.71) |  |  |  |
|  | 3 months | | 4.45 (4.06 to 4.83) | 5.04 (4.65 to 5.42) |  |  |  |
|  | Change | | −0.40 (−0.74 to −0.06) | −0.32 (−0.62 to −0.02) |  |  |  |
| **Anxiety (HADS-A^c^)** | | | | | 0.077 (−0.69 to 0.93) | .86 | .013 |
|  | Baseline | | 6.82 (6.11 to 7.52) | 7.89 (7.08 to 8.71) |  |  |  |
|  | 3 months | | 6.20 (5.51 to 6.90) | 7.32 (6.52 to 8.12) |  |  |  |
|  | Change | | −0.61 (−1.12 to −0.08) | −0.58 (−1.12 to −0.03) |  |  |  |
| **Depression (HADS-D^d^)** | | | | | −0.90 (−1.73 to −0.02) | .03 | −.158 |
|  | Baseline | | 6.67 (5.92 to 7.42) | 7.17 (6.49 to 7.85) |  |  |  |
|  | 3 months | | 5.46 (4.81 to 6.11) | 6.85 (6.14 to 7.56) |  |  |  |
|  | Change | | −1.83 (−2.75 to −0.90) | −0.89 (−1.78 to −0.01) |  |  |  |
| **Self-regulatory fatigue (SRF-18^e^)** | | | | | −2.76 (−5.07 to −0.73) | .008 | −.181 |
|  | Baseline | | 53.53 (51.57 to 55.49) | 55.31 (53.20 to 57.42) |  |  |  |
|  | 3 months | | 50.45 (48.51 to 52.38) | 54.94 (52.95 to 56.93) |  |  |  |
|  | Change | | −3.08 (−4.40 to −1.77) | −0.37 (−1.82 to 1.09) |  |  |  |
| **HRQoL^f^ (RAND-36^g^)** | | | | | | | |
|  | **Physical functioning** | | | | 2.19 (−2.11 to 6.30) | .32 | .071 |
|  |  | Baseline | 57.29 (52.55 to 62.03) | 54.21 (50.15 to 58.26) |  |  |  |
|  |  | 3 months | 61.83 (57.04 to 66.63) | 56.71 (52.58 to 60.84) |  |  |  |
|  |  | Change | 4.54 (1.81 to 7.27) | 2.50 (−0.46 to 5.46) |  |  |  |
|  | **Role physical** | | | | −5.18 (−13.28 to 2.51) | .24 | −.083 |
|  |  | Baseline | 20.87 (14.69 to 27.05) | 11.25 (7.55 to 14.95) |  |  |  |
|  |  | 3 months | 26.15 (19.55 to 32.75) | 19.58 (14.02 to 25.15) |  |  |  |
|  |  | Change | 5.28 (−0.21 to 10.76) | 8.33 (2.32 to 14.35) |  |  |  |
|  | **Bodily pain** | | | | 1.54 (−3.01 to 5.96) | .50 | .047 |
|  |  | Baseline | 27.73 (24.70 to 30.76) | 26.85 (24.21 to 29.50) |  |  |  |
|  |  | 3 months | 30.89 (27.33 to 34.46) | 28.96 (25.54 to 32.38) |  |  |  |
|  |  | Change | 3.17 (−0.16 to 6.50) | 2.10 (−0.72 to 4.93) |  |  |  |
|  | **General health** | | | | 3.04 (−0.74 to 6.80) | .11 | .111 |
|  |  | Baseline | 38.21 (34.37 to 42.05) | 36.54 (32.97 to 40.11) |  |  |  |
|  |  | 3 months | 42.71 (39.02 to 46.40) | 38.25 (34.70 to 41.80) |  |  |  |
|  |  | Change | 4.50 (1.93 to 7.06) | 1.71 (−0.79 to 4.20) |  |  |  |
|  | **Vitality** | | | | 4.25 (0.15 to 8.16) | .05 | .136 |
|  |  | Baseline | 27.80 (24.18 to 31.42) | 25.25 (21.63 to 28.87) |  |  |  |
|  |  | 3 months | 34.40 (30.40 to 38.40) | 27.17 (23.41 to 30.92) |  |  |  |
|  |  | Change | 6.61 (3.61 to 9.60) | 1.92 (−0.82 to 4.66) |  |  |  |
|  | **Social functioning** | | | | 2.68 (−3.80 to 8.84) | .45 | .055 |
|  |  | Baseline | 47.71 (42.90 to 52.51) | 46.67 (42.31 to 51.02) |  |  |  |
|  |  | 3 months | 54.82 (49.98 to 59.65) | 51.04 (46.12 to 55.97) |  |  |  |
|  |  | Change | 7.11 (2.85 to 11.37) | 4.38 (−0.28 to 9.03) |  |  |  |
|  | **Role emotional** | | | | 7.01 (−5.93 to 19.88) | .32 | .068 |
|  |  | Baseline | 48.32 (39.81 to 56.82) | 52.22 (44.04 to 60.40) |  |  |  |
|  |  | 3 months | 57.80 (49.55 to 66.04) | 54.44 (46.11 to 62.78) |  |  |  |
|  |  | Change | 9.48 (0.20 to 18.76) | 2.22 (−7.62 to 12.06) |  |  |  |
|  | **Mental health** | | | | 3.03 (−1.33 to 7.63) | .14 | .105 |
|  |  | Baseline | 67.08 (63.80 to 70.37) | 64.50 (61.20 to 67.80) |  |  |  |
|  |  | 3 months | 69.32 (65.77 to 72.88) | 63.30 (59.66 to 66.94) |  |  |  |
|  |  | Change | 2.24 (−0.64 to 5.12) | −1.20 (−3.66 to 1.26) |  |  |  |
| **Pain catastrophizing (PCS^h^)** | | | | | | | |
|  | **Rumination** | | | | −0.61 (−1.38 to .19) | .16 | −.093 |
|  |  | Baseline | 7.26 (6.53 to 7.99) | 8.11 (7.42 to 8.80) |  |  |  |
|  |  | 3 months | 6.41 (5.70 to 7.12) | 7.86 (7.16 to 8.55) |  |  |  |
|  |  | Change | −0.84 (−1.44 to −0.25) | −0.25 (−0.88 to 0.38) |  |  |  |
|  | **Magnification** | | | | −0.18 (−0.67 to 0.32) | .50 | −.044 |
|  |  | Baseline | 3.23 (2.77 to 3.69) | 3.67 (3.17 to 4.16) |  |  |  |
|  |  | 3 months | 2.94 (2.51 to 3.36) | 3.61 (3.16 to 4.05) |  |  |  |
|  |  | Change | −0.29 (−0.65 to 0.064) | −0.06 (−0.46 to 0.34) |  |  |  |
|  | **Helplessness** | | | | −0.32 (−1.34 to 0.70) | .54 | −.041 |
|  |  | Baseline | 8.20 (7.32 to 9.08) | 9.37 (8.42 to 10.32) |  |  |  |
|  |  | 3 months | 7.79 (6.88 to 8.70) | 9.18 (8.31 to 10.05) |  |  |  |
|  |  | Change | −.41 (−1.14 to 0.31) | −0.18 (−0.89 to 0.53) |  |  |  |
|  | **PCS total** | | | | 1.03 (−.54 to 2.45) | .19 | .101 |
|  |  | Baseline | 18.69 (16.91 to 20.47) | 21.14 (19.24 to 23.04) |  |  |  |
|  |  | 3 months | 17.14 (15.33 to 18.95) | 20.65 (18.90 to 22.40) |  |  |  |
|  |  | Change | −0.69 (−0.39 to 1.77) | −0.26 (−1.08 to 0.57) |  |  |  |
| **Chronic pain acceptance (CPAQ^i^)** | | | | | | | |
|  | **Willingness** | | | | −0.32 (−1.34 to 0.70) | .54 | .039 |
|  |  | Baseline | 13.63 (13.06 to 14.20) | 13.69 (13.15 to 14.23) |  |  |  |
|  |  | 3 months | 13.78 (13.23 to 14.33) | 13.63 (13.15 to 14.11) |  |  |  |
|  |  | Change | 0.15 (−0.50 to 0.80) | −0.06 (−0.58 to 0.47) |  |  |  |
|  | **Activity engagement** | | | | 0.079 (−0.06 to 1.65) | .08 | .128 |
|  |  | Baseline | 13.82 (13.07 to 14.56) | 13.85 (13.21 to 14.49) |  |  |  |
|  |  | 3 months | 14.36 (13.73 to 14.99) | 13.65 (13.04 to 14.26) |  |  |  |
|  |  | Change | 0.54 (−0.07 to 1.15) | −0.20 (−0.73 to 0.33) |  |  |  |
|  | **CPAQ total** | | | | 1.03 (−0.47 to 2.43) | .16 | .101 |
|  |  | Baseline | 27.45 (26.26 to 28.64) | 27.54 (26.53 to 28.56) |  |  |  |
|  |  | 3 monts | 28.14 (27.19 to 29.09) | 27.28 (26.33 to 28.23) |  |  |  |
|  |  | Change | 0.69 (−0.39 to 1.77) | −0.26 (−1.08 to 0.57) |  |  |  |

^a^BPI: Brief Pain Inventory.

^b^Subscale of the BPI (score range 0-10; a higher score indicates higher interference in life).

^c^HADS-A: Hospital Anxiety and Depression Scale-Anxiety subscale. The score range is 0 to 21; a higher score indicates a higher degree of anxiety.

^d^HADS-D: Hospital Anxiety and Depression Scale-Depression subscale. The score range is 0 to 21; a higher score indicates a higher degree of depression.

^e^SRF-18: Self-Regulatory Fatigue 18. The score range is 18 to 90; a higher score indicates higher self-regulatory fatigue.

^f^HRQoL: health-related quality of life.

^g^RAND-36: SF-36 Short Form Health Survey. The score range is 0 to 100; a higher score indicates higher emotional well-being.

^h^PCS: Pain Catastrophizing Scale. The score range is 0 to 52; a higher score indicates higher catastrophizing.

^i^CPAQ: Chronic Pain Acceptance Questionnaire. The score range is 0 to 52; a higher score indicates a higher acceptance of pain.
